# Supplementary material for: 3D printed biomimetic cochleae and machine learning co-modelling provides clinical informatics for cochlear implant patients
Source: Nat Commun. 2021 Oct 29;12:6260. doi: 10.1038/s41467-021-26491-6 (PMC8556326; doi:10.1038/s41467-021-26491-6)
Supplement: Supplementary file 1 — Supplementary information [file 41467_2021_26491_MOESM1_ESM.pdf]

Supplementary information

Title: 3D printed biomimetic cochleae and machine learning co-modelling provides clinical informatics for cochlear implant patients

Authors

Iek Man Lei[1,2], Chen Jiang[3,1,4], Chon Lok Lei[5,6], Simone Rosalie de Rijk[3], Yu Chuen Tam[7], Chloe Swords[8], Michael P.F. Sutcliffe[1], George G. Malliaras[1], Manohar Bance[3,*], Yan Yan Shery Huang[1,2,*]

Affiliations

[1]*Department of Engineering, University of Cambridge, United Kingdom*

[2]*The Nanoscience Centre, University of Cambridge, United Kingdom*

[3]*Department of Clinical Neurosciences, University of Cambridge, United Kingdom*

[4]*Department of Electronic Engineering, Tsinghua University, Beijing 100084, China*

[5]*Institute of Translational Medicine, Faculty of Health Sciences, University of Macau, Macau*

[6]*Department of Computer Science, University of Oxford, United Kingdom*

[7]*Emmeline Centre for Hearing Implants, Addenbrookes Hospital, Cambridge, United Kingdom*

[8]*Department of Physiology, Development and Neurosciences, Cambridge, United Kingdom*

*Corresponding authors: yysh2@cam.ac.uk and mlb59@cam.ac.uk. These authors jointly supervised this work.

Table of contents

I. Finite element modelling of cochlear stimulation 4

Supplementary Fig.1| Finite element modelling of cochlear stimulation. 4

II. Designable electro-mimetic bone matrices to mimic the resistivity of cochlear bone 7

Supplementary Fig.2| Reported resistivities of skull/cortical bones in literature 7

Supplementary Fig.3| Interconnected microchannel network in the electro-mimetic bone matrices for tuneable resistivity 8

Supplementary Fig.4| Equivalent circuit model of the EIS measurements. 10

Supplementary Fig.5| Electrical resistivity and appearance of 3D printed cochlear models made of different materials. Long-lasting properties and EFI repeatability of the biomimetic cochleae. 11

Supplementary Note 1| Mechanics analysis of the deformation followed by CI electrode array insertions. 13

III. Patients' clinical cochlear CT scans 14

Supplementary Fig.6| Example showing 2 patients' clinical cochlear CT scans acquired in the pre-operative assessment. 14

IV. 3D printed biomimetic cochleae 15

Supplementary Table 1| Definitions and the ranges of the model descriptors investigated in this study in comparison with the reported variation in human cochleae. 15

Supplementary Fig.7| Biomimetic cochleae capturing the electrode positions in patient cochlea. 16

V. 3PNN – '3D printing and neural network co-modelling' 17

Supplementary Note 2| Rationale for using neural network as a modelling approach compared to existing modelling approaches. 17

Supplementary Fig.8| Lumped parameter model for modelling volume conduction in cochleae with our patients' data. 19

Supplementary Fig.9| Hyperparameter tuning of 3PNN. 20

Supplementary Table 2| Input values of the stimulating and the recording electrode positions of different electrode types used in 3PNN. 21

Supplementary Fig.10| Applicability of 3PNN on different electrode types. 22

Supplementary Fig.11| Validation of forward-3PNN. 23

Supplementary Fig.12| Predictive performance of 3PNN against the EFI profiles within patient population. 24

Supplementary Table 3| 3PNN performance compared with the MAPE obtained from the population mean EFI. 25

Supplementary Fig.13| Validation of inverse-3PNN. 26

Supplementary Fig.14| Example showing the trend of the predicted EFI profiles along the 5 model descriptors. 27

Supplementary Fig.15| Example of the power-law fitting analysis of EFIs. 28

Supplementary Fig.16| Goodness-of-fit test to evaluate the choice of the fitting forms 29

Supplementary Fig.17| First-order and total-order Sobol sensitivity indices of the $|Slope|_{x=1mm}$ and baseline coefficient for different model descriptors 30

Supplementary Table 4| Second-order Sobol sensitivity indices of the $|Slope|_{x=1mm}$ and baseline coefficient. 31

Supplementary Table 5| First-order Sobol sensitivity indices of EFIs for different model descriptors 32

Supplementary Fig.18| The predicted distributions of the model descriptors of subjects 1^{1J} and 2^{1J}, and the selected parameters for fabricating on-demand patient-specific biomimetic cochleae. 33

Supplementary Fig.19| 3PNN estimating patient-specific resistivity of the cochlear tissue 33

Supplementary Fig.20| Sensitivity analyses assessing the effect of the potential sources of uncertainty on EFIs. 34

Supplementary Table 6| Potential uncertainties in 3PNN, and their estimated effect on off-stimulation EFIs. 35

Supplementary Methods 36

Finite element modelling of the intracochlear voltage distribution in cochlea 36

Supplementary Fig.21| Domain properties of the COMSOL models. 37

Embedded 3D printing of linear electro-mimetic bone matrix models 37

Supplementary Fig.22| Correlation of the actual dimensions of the model descriptors measured from μ-CT and the process parameters of the robotic 3D printer. 38

Supplementary Table 7| Specifications of the 82 biomimetic cochleae used in 3PNN training. 39

Supplementary Fig.23| Photo demonstrating the insertion of a CI electrode array in a biomimetic cochlea during EFI measurements. 40

Supplementary Table 8| The final MAPE thresholds used in the inverse predictions. 41

Abbreviation Table 42

Supplementary References 43

I. Finite element modelling of cochlear stimulation

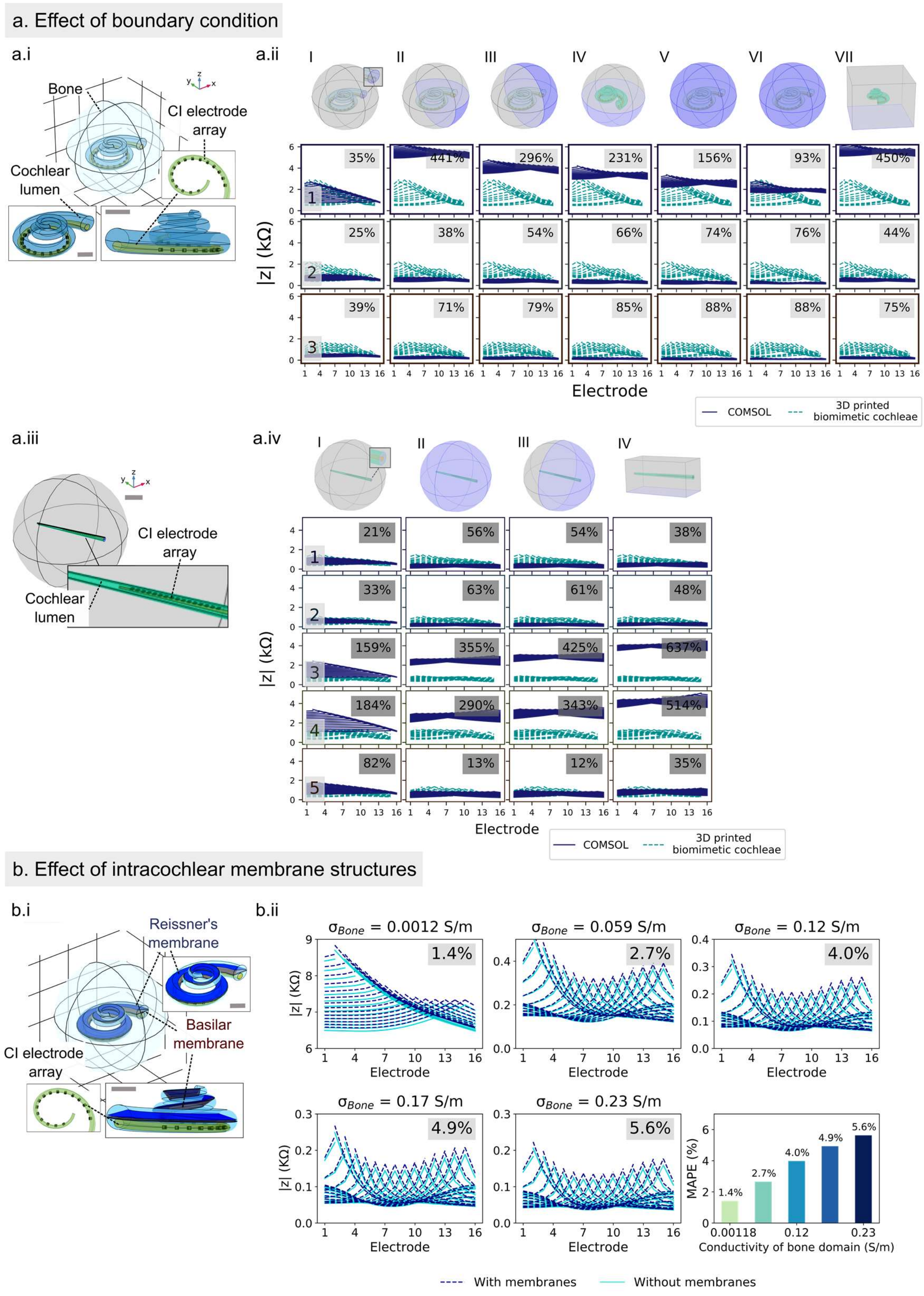

Supplementary Fig.1| Finite element modelling of cochlear stimulation.

a, The effect of the choice of the boundary conditions on finite element modelling of cochlear stimulation

i. A finite element model of a simplified spiral cochlea without the intracochlear membrane structures. Scale bar = 2 mm. Detailed method of the development of the finite element models can be found in Supplementary Methods.

ii. Off-stimulation EFI profiles simulated with the common choices of boundary conditions used in literature, in comparison with the experimental results acquired from the corresponding 3D printed cochlear models that have the same electroanatomical model descriptors as the COMSOL models. The values at the upper right of the graphs indicate the MAPEs (median absolute percentage error) between the simulated EFIs and the experimental EFIs. 3 models are examined here. They have the same geometric features (BL_d = 2.38 mm, Tr_a = 0.59, W_c = 10.5 mm, h_c = 4.4 mm) but different matrix resistivities (model 1: 23.4 kΩcm, model 2: 1.9 kΩcm and model 3: 0.7 kΩcm). BL_d = basal lumen diameter, Tr_a = taper ratio, W_c = cochlear width and h_c = cochlear height. Detailed descriptions of the model descriptors can be found in Supplementary Table 1. The FEM boundary conditions studied here are ground at **(I)** the cochlear lumen opening, **(II)** a quarter of the outer surface, which is near the lumen opening, of the 8 mm radius sphere. **(III)** half of the outer surface of the 8 mm radius sphere, **(IV)** the bottom half of the outer surface of the 8 mm radius sphere, **(V)** the outer surface of the 8 mm radius sphere, **(VI)** the outer surface of an infinitely surrounding sphere, and **(VII)** the bottom surface of a 20x20x15 mm cuboid, as highlighted in blue. In experiments, the extracochlear ground was placed underneath the 3D printed models, similar to the condition VII in FEM.

The choice of the boundary condition was known to be undetermined in simulating cochlear stimulation via FEM[1]. In monopolar cochlear stimulation, the return electrode lies outside the physical domain of the model, hence, there is no boundary condition that can perfectly match the *in vivo* situation[1]. As shown in this graph, the simulated FEM profile is greatly affected by the choice of the boundary condition. We found that placing the ground at the lumen opening in FEM (condition I), yields the EFIs most matching to the experimental EFIs. However, the resemblance is still not ideal. In addition, even condition VII in FEM exactly mimics the five electroanatomical features of the 3D printed models and the grounding condition employed in experiments, FEM cannot reproduce the results of the physical models due to the undetermined modelling parameters. The results highlight the benefits of using physical models, which bypass the sensitivity of the choice of boundary conditions and the explicit descriptions of physical laws. Further discussion on the effect of the boundary conditions on EFI can be found in the work by Wong et al. (2016)[1].

iii. A finite element model of a linear uncoiled cochlea without the intracochlear membrane structures. Scale bar = 10 mm.

iv. Off-stimulation EFI profiles simulated using four different choices of boundary condition, in comparison with the experimental results acquired from the corresponding 3D printed linear cochlear models that have the same electroanatomical model descriptors as the COMSOL models. 4 linear models were examined here. The fabrication method of the 3D printed linear models was similar to that of the spiral biomimetic cochlear models (Supplementary Methods). They have the following electroanatomical features – Model 1: BL_d = 2.38 mm, Tr_a = 1, L_c = 40.6 mm, and R_m = 23.4 kΩcm; Model 2: BL_d = 2.38 mm, Tr_a = 0.79, L_c = 40.6 mm, and R_m = 1.9 kΩcm; Model 3: BL_d = 2.38 mm, Tr_a = 0.79, L_c = 40.6 mm, and R_m = 1.9 kΩcm; Model 4: BL_d = 2.5 mm, Tr_a = 0.59, L_c = 40.6 mm, and R_m = 3.6 kΩcm. BL_d = basal lumen diameter, Tr_a = taper ratio, L_c = length of the uncoiled cochlear lumen, and R_m = matrix resistivity. The values at the upper right indicate the MAPEs between the FEM simulated EFIs and the experimental EFIs. The boundary condition examined here are ground at **(I)** the cochlear lumen opening, **(II)** the outer surface of the 30 mm radius sphere, **(III)** half of the outer surface of the 30 mm radius sphere, and **(IV)** the bottom surface of a 40.6x15x15 mm cuboid, as indicated in blue.

Similar to the above results obtained from spiral cochlear model, condition I is more likely to yield the EFIs most matching to the experimental EFIs, but large discrepancy between the simulated EFI and the experimental EFI (MAPE between ~20% to ~180% depending on the model features) is observed.

b, The effect of the intracochlear membrane structures on finite element modelling of cochlear stimulation

i. A finite element model of a cochlea with the Reissner's membrane and the Basilar membrane. Scale bar = 2 mm.

ii. Simulated off-stimulation EFIs generated from the membranous cochlear model (Supplementary Fig.1b(i)) and from the simplified cochlear model (Supplementary Fig.1a(i)) with different bone domain conductivities, σ_{bone}. The models have the following geometric features – BL_d = 2.1 mm, Tr_a = 0.62, W_c = 10.5 mm and h_c = 4.4 mm. In the simulations here, the ground was set to be infinitely far away from the cochlea. σ_{bone} was varied from 0.0012 to 0.23 S/m to consider the wide variation in the reported conductivity of skulls and cortical bones in live human, animals and cadavers (Supplementary Fig.2a)[2–13]. The values at the upper right of the graphs indicate the MAPE between the simulated EFIs of the membranous cochlear model and the simplified model. As shown in the graph, MAPE increases with σ_{bone}. Hence, the largest MAPE between the two models happened at σ_{bone} = 0.23 S/m with a MAPE only ~ 6%.

.

II. Designable electro-mimetic bone matrices to mimic the resistivity of cochlear bone

a

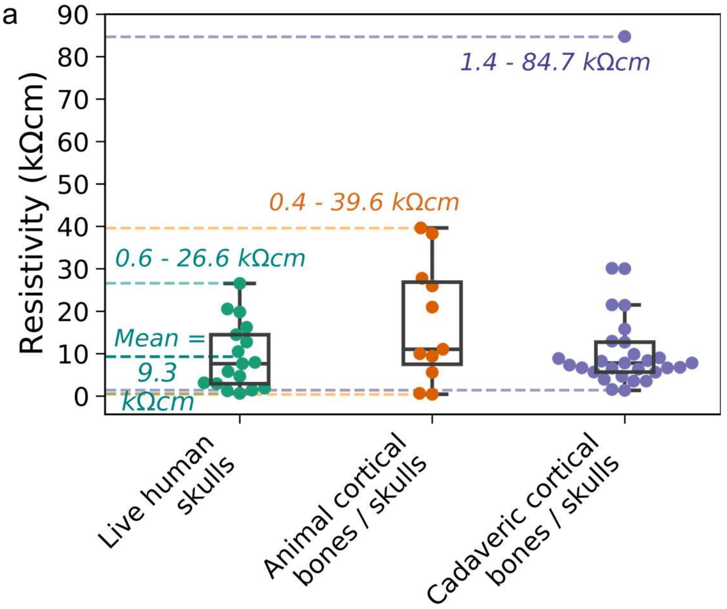

b

Species	Site	Resistivity (kΩcm)	Sources
Live adult human	Skull	5.8 – 26.6	Tang, et al., (2008)[2]
Live adult human	Skull	4.7 – 20.5	Akhtari, et al. (2002)[3]
Live adult human	Skull	1.2 – 3.1	Hoekema, et al. (2003)[6]
Live adult human	Skull	7.6	Oostendorp, et al. (2000)[7]
Live adult human	Skull	~0.6	Fernandez-Corazza, et al (2018)[5]
Cadaver	Skull (saline soaked)	12.9 – 84.7	Akhtari, et al. (2000)[8]
Cadaver	Skull (Saline + formalin soaked)	4.7	Hoekema, et al. (2003)[6]
Cadaver	Skull (saline soaked)	1.4 – 21.4	Law, et al. (1993)[9]
Cadaver	Skull (saline soaked)	6.7	Oostendorp, et al. (2000)[7]
Cadaver	Cortical bone (saline soaked)	1.6 – 21.5	Saha, et al. (1992)[10]
Gerbil specimen	Cochlea	0.4	Micco, et al. (2006)[11]
Adult bovine	Cortical tibia (saline soaked)	5.6 – 39.6	Balmer, et al. (2018)[12]
Guinea pig	n/a	0.6	Frijns, et al. (1995)[13] Briaire, et al. (2000)[4]

Supplementary Fig.2| Reported resistivities of skull/cortical bones in literature.

a, Resistivities of live human, animal and human cadaveric cortical bones or skulls reported in literature[2–13].

b, Table summarised the reported resistivities of cortical bones or skulls obtained from various species and sites.

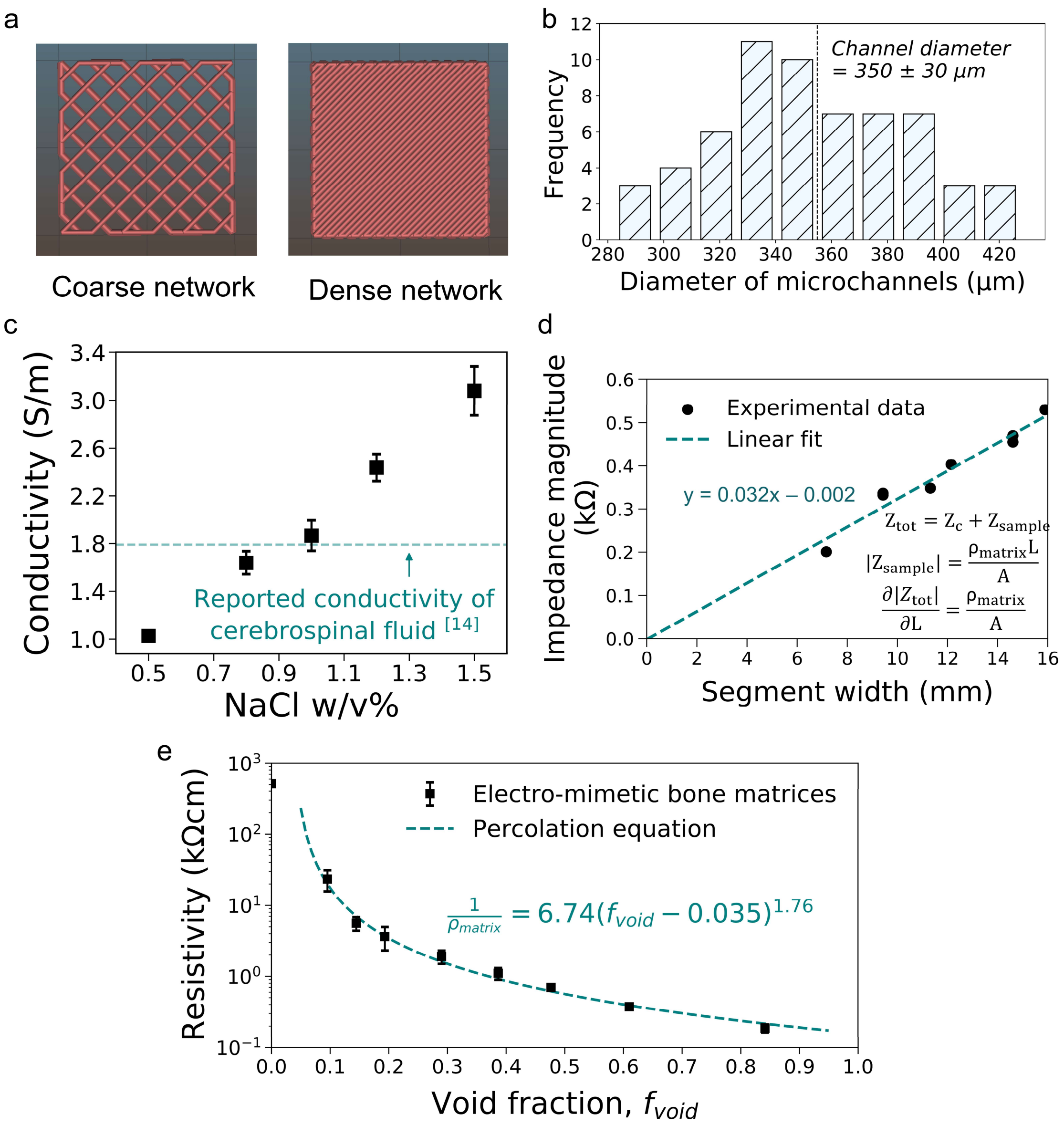

Supplementary Fig.3| Interconnected microchannel network in the electro-mimetic bone matrices for tuneable resistivity.

a, Examples of a coarse network and a dense network designed on Slic3R by varying the fill density setting for controlling the density of the interconnected microchannel network in the matrix and, therefore, the resistivity of the electro-mimetic bone matrices.

b, A histogram of the diameter of the microchannels in the electro-mimetic bone matrices. (7 independent samples with different densities of microchannels were fabricated and the diameter of the microchannel was measured at 8 – 9 different locations of each sample. In total, 61 measurements were made with the 7 samples, n = 61). The microchannels were found to have a mean diameter of 350 ± 30 µm, measured from the microscopic images of the matrices.

c, Electrical conductivity of NaCl solutions at various concentrations at ambient temperature. The measurement at each concentration was independently repeated 4 times (n = 4) using a freshly prepared solution in each measurement. Data are presented as mean values ± SD. The voids (including both microchannels and the cochlea-shaped lumen) in the electro-mimetic bone matrices were filled with a 1 w/v% NaCl solution. The rationale for using a 1 w/v% NaCl solution is that 1 w/v% NaCl solution at ambient temperature has the most similar conductivity to human cerebrospinal fluid, which is 1.79 S/m at body temperature[14]. As perilymph has a similar ionic composition to cerebrospinal fluid[15], we used the conductivity of the cerebrospinal fluid to approximate the conductivity of perilymph. Hence, using a 1 w/v% NaCl solution not only tunes the resistivity of the electro-mimetic bone matrix but also imitates the electrical properties of the perilymph inside human cochleae.

d, Example showing the use of 'transmission-line' method to determine the resistivity of the electro-mimetic bone matrix ρ_{matrix} associated with the frequency range that results in the plateau impedance (~ 300 Hz – 100 kHz), where Z_{tot} = plateau value of the total impedance, Z_c = contact impedance, Z_{sample} = impedance of the sample, L = width of the segment of the sample, A = area of the electrode plate in contact with the sample, and ρ_{matrix} = resistivity of the electro-mimetic bone matrix sample.

e, Electrical resistivity (plateau value) of the electro-mimetic bone matrices vs the volumetric void fraction f_{void} of the matrices. (n = 3; Each resistivity measurement was repeated 3 times using independent samples). Data are presented as mean values ± SD.

f_{void} was estimated using the following equation $f_{void} = (A_{channel} \times d_{printing\ path}) / V_{matrix}$, where $A_{channel}$ = average cross-sectional area of the microchannel derived from the mean diameter of the microchannels (Supplementary Fig.3b), $d_{printing\ path}$ = total distance of the printing path and V_{matrix} = total volume of the matrix.

To describe the correlation between the resistivity of the electro-mimetic bone matrix ρ_{matrix} and f_{void}, we assume that the electro-mimetic bone matrix behaves as a conductor-insulator composite. The resistivity of such composite is given by a percolation equation with the form $1/\rho_{matrix} = \alpha(f_{void} - f^*)^{\beta}$, where α and β are fitting coefficients, and f^* is known as the percolation threshold (the minimum volumetric fraction of the saline-filled void for electrical current to percolate from one side to the other[16]). With the assumption that an electrical percolation network will first be established when there are two diagonal channels in the matrix, we found that f^* = 0.035. The fitting equation was found to be $1/\rho_{matrix} = 6.74(f_{void} - 0.035)^{1.76}$ after experimental data fitting.

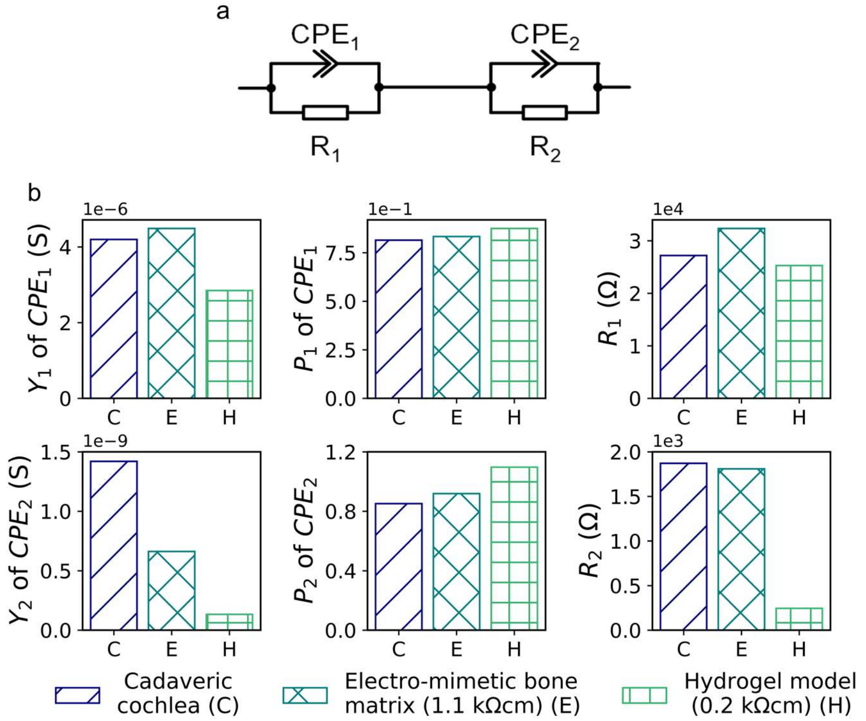

Supplementary Fig.4| Equivalent circuit model of the EIS measurements.

a, Schematic of the equivalent circuit model for analysing the EIS (electrochemical impedance spectroscopy) measurements of a cadaveric cochlea in a head, a hydrogel 3D printed model and an electro-mimetic bone matrix model (biomimetic cochlea). We employed a similar equivalent circuit model from our previous study[17], which consists of a parallel circuit of a resistor (R_1) and a constant phase element (CPE_1) in series with another parallel circuit of a resistor (R_2) and a constant phase element (CPE_2). The complex impedance of a *CPE* can be expressed as $\dot{Z}_{CPE}(\omega) = \frac{1}{Y(j\omega)^P}$, where *Y* is the admittance magnitude at 1 rad/s and *P* is the parameter for the constant phase ($\theta = -90p^o$).

b, The extracted parameters of the resistors *(R1, R2)* and the CPEs (CPE_1, CPE_2) of the equivalent circuit model. As shown here, the CPE_1 and R_1 parameters are in the same order of magnitudes for all models, but the electro-mimetic bone matrix model has a more similar CPE_2 and R_2 parameters to the cadaveric cochlea, in contrast to the parameters obtained from the hydrogel model. Our previous study found that the CPE_2 and R_2 had strong correlation to cochlear structures and their electrical impedances, while the CPE_1 and R_1 are related to the current pathway via temporal bones to the ground[17]. Therefore, the CPE_2 and R_2 parameters are of interest here for evaluating the similarity in their impedance properties. It should be noted that Y_2 *of* CPE_2 is affected by bone compositions, cadaver age, anatomical geometries, CI insertion depth etc[17–19]. In our previous study, we found that Y_2 varies from 0.5 to 3 nS due to the variation in cadaveric cochleae[17]. This range is of a similar magnitude compared to the Y_2 obtained from the electro-mimetic bone matrix model.

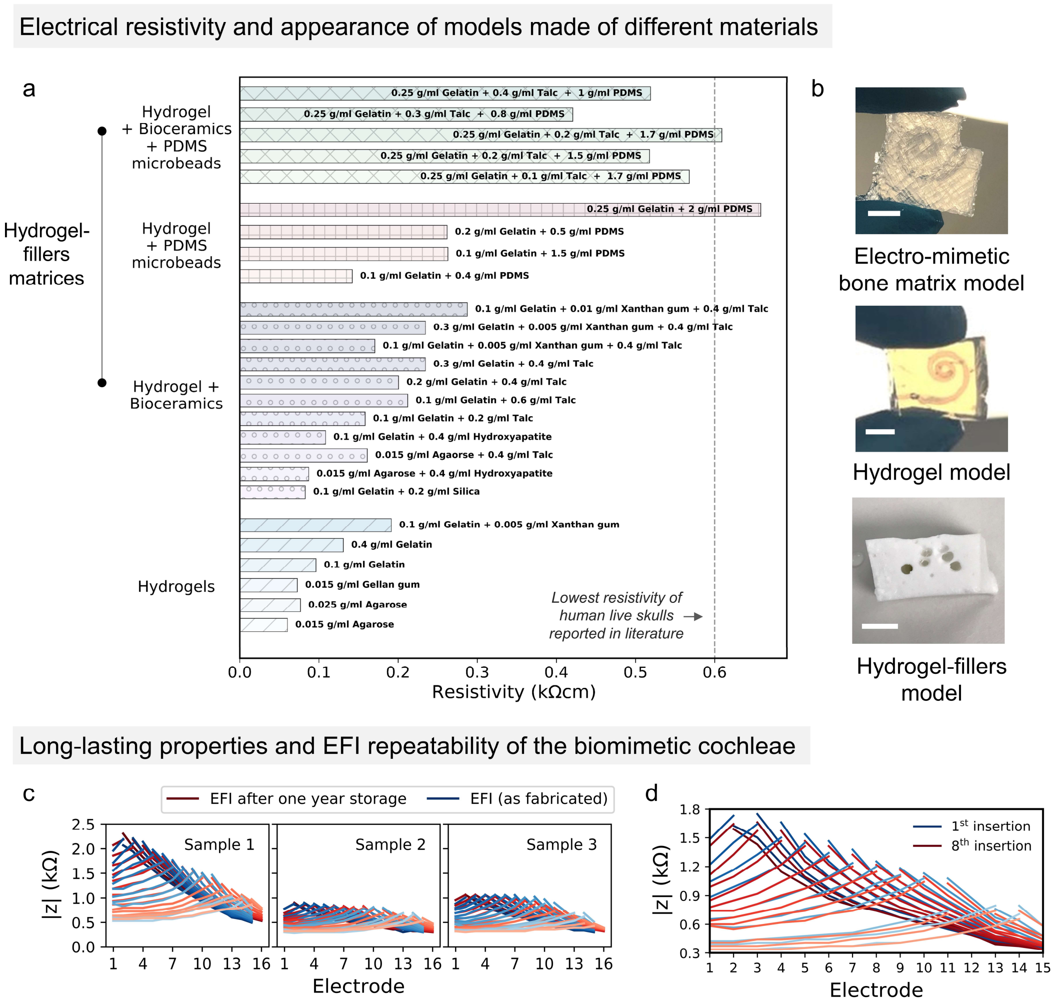

Supplementary Fig.5| Electrical resistivity and appearance of 3D printed cochlear models made of different materials. Long-lasting properties and EFI repeatability of the biomimetic cochleae.

- **a,** Resistivity (plateau value) of the hydrogel matrices and hydrogel-fillers matrices (hydrogel-bioceramics matrices, hydrogen-PDMS microbeads matrices and hydrogel-bioceramics-PDMS microbeads matrices) tested in this study. All the polymers and the fillers (bioceramics or PDMS microbeads) were dissolved or dispersed in a 1 w/v% NaCl solution. The hydrogel and hydrogel-fillers matrices exhibit poor resistivity tunability. We found that the hydrogel-based matrices cannot imitate the full reported range of the resistivity of human live skulls, and can only achieve the lowest resistivity reported in literature. In addition, these matrices are soft and fragile, which will lead to inconsistent electrical property over time.

- **b,** Appearance of 3D printed cochlear models made of an electro-mimetic bone matrix, a hydrogel, and a hydrogel-fillers matrix. The translucent appearance of our electro-mimetic bone matrix model may help direct visualisation of the insertion depth of the CI electrode array inside its cochlear lumen, in contrast to the opaque appearance associated with a hydrogel-fillers model. Scale bar = 5 mm.

c, EFIs measured in the same electro-mimetic bone matrix cochlear model before and after a year storage. Their high similarity indicates the long-lasting property of the PDMS-based biomimetic cochleae.

d, EFIs measured in the same electro-mimetic bone matrix cochlear model after multiple CI insertions. No significant change is observed, illustrating the reusable property of our PDMS-based electro-mimetic bone matrix model. This also confirms that the applied force during CI insertions will not impose any significant deformation to the shape of the cochlear lumen of the model.

Supplementary Note 1| Mechanics analysis of the deformation followed by CI electrode array insertions.

Global and local deformation behaviours of the biomimetic cochlea were calculated. We assume that the matrix is an isotropic solid, and the force applied to the model during insertion *F* is 0.004 N, which is the reported average insertion force by the surgeons[20].

Global deformation

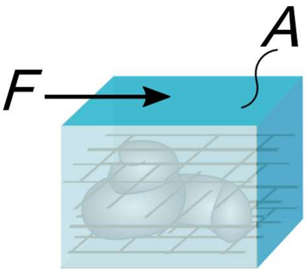

Given that shear stress τ and shear strain γ are expressed as $\tau = \frac{F}{A}$ and $\gamma = \frac{\tau}{G}$, therefore

$$\gamma = \frac{F}{AG}$$

where *F* = insertion force, *G* = shear modulus and *A* = cross-sectional area of the model parallel to the applied force, which is assumed to be 15x15 mm^2.

Assuming the matrix is an isotropic solid, the following equations are obtained, where *E* is the Young's modulus (~ 10^6 Pa as shown in Fig.3) and *v* is the Poisson's ratio of the matrix.

$$G = \frac{E}{2(1+v)} \approx \frac{E}{3}$$

The global shear deformation of the matrix caused by CI electrode array insertion was therefore estimated using

$$\gamma = \frac{3F}{AE} = \frac{3*0.004}{0.015^2*10^6} = 10^{-5}.$$

Local deformation

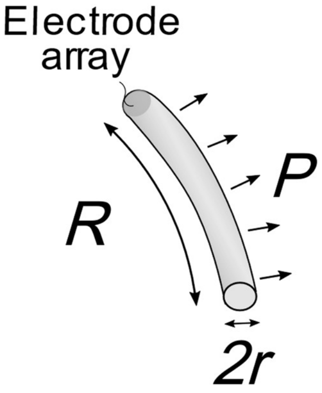

The pressure *P* exerted on the lumen wall of the biomimetic cochlea is assumed to follow

$$P = \frac{F}{A} \approx \frac{F}{2rR}$$

where *r* = cross-sectional radius of the electrode array (0.3 mm, the average radius of the HiFocus™ 1J electrode (CI[1J]) electrode array[21]), *R* = length of the electrode array (25 mm[21]) and *F* = insertion force.

As an approximation, the local normal strain ε is estimated using the below expression.

$$\varepsilon \approx \frac{P}{E}$$

$$\varepsilon \approx \frac{F}{2rRE} = \frac{0.004}{2*0.003*0.025*10^6} = 10^{-5}.$$

Therefore, as the global shear strain and local normal strain caused by CI electrode array insertions are insignificant (~10^{-5}), we expect that the insertion of CI electrode array into the PDMS-based biomimetic cochlea will not impose any significant deformation to the matrix.

III. Patients' clinical cochlear CT scans

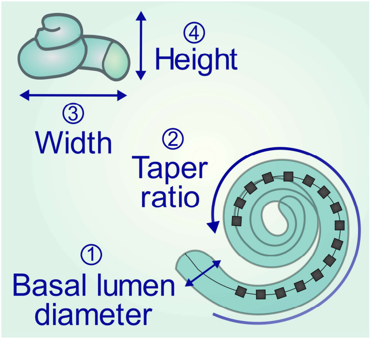

Subject 4^{SlimJ}

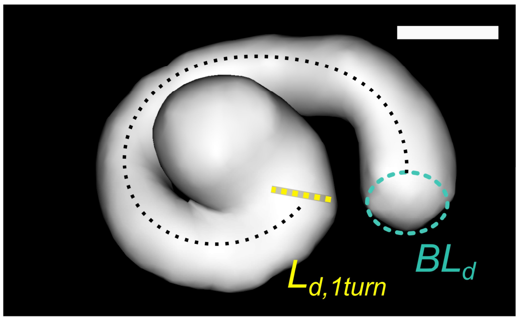

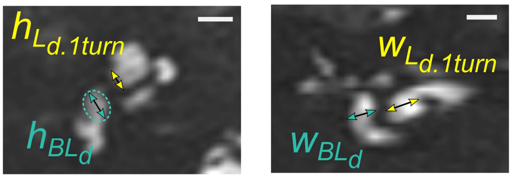

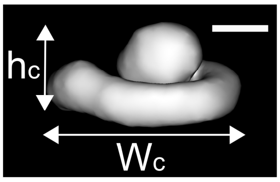

Height, $\mathbf{h_c}$ = 4.7 mm

Width, $\mathbf{W_c}$ = 8.8 mm

Subject 6^{SlimJ}

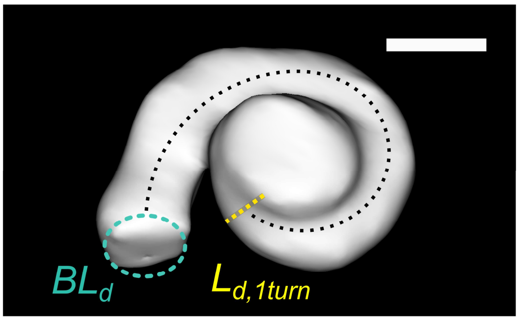

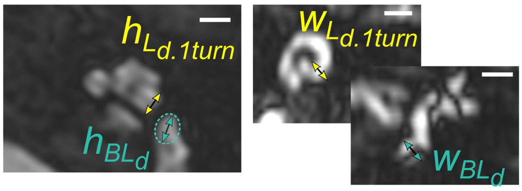

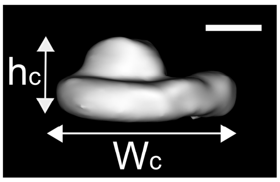

Height, $\mathbf{h_c}$ = 4.1 mm

Width, $\mathbf{W_c}$ = 8.1 mm

Basal lumen diameter, $\mathbf{BL_d}$

$$= \frac{h_{BL_d}+W_{BL_d}}{2} = \frac{(2.36+2.4)}{2} = 2.4 \text{ mm}$$

$$L_{d,1turn} = \frac{h_{L_{d,1turn}} + W_{L_{d,1turn}}}{2} = \frac{(1.62 + 2.56)}{2} = 2.1 \text{ mm}$$

$$\textbf{Taper ratio, } \mathbf{Tr_a} = \frac{L_{d,1turn}}{BL_d} = \frac{2.1}{2.4} = 0.9 \text{ mm}$$

Basal lumen diameter, $\mathbf{BL_d}$

$$= \frac{h_{BL_d}+W_{BL_d}}{2} = \frac{(2.4+2)}{2} = 2.2 \text{ mm}$$

$$L_{d,1turn} = \frac{h_{L_{d,1turn}} + W_{L_{d,1turn}}}{2} = \frac{(1.88 + 2)}{2} = 1.9 \text{ mm}$$

$$\textbf{Taper ratio, } \mathbf{Tr_a} = \frac{L_{d,1turn}}{BL_d} = \frac{1.9}{2.2} = 0.9 \text{ mm}$$

Supplementary Fig.6| Example showing 2 patients' clinical cochlear CT scans acquired in the pre-operative assessment.

The four geometric descriptors, basal lumen diameter (BL_d), taper ratio (Tr_a), cochlear height (h_c) and cochlear width (W_c), were measured and adopted in the 3PNN analysis. Detailed definitions of the geometric descriptors can be found in Supplementary Table 1. Scale bar = 2.5 mm. $L_{d,1turn}$ = cochlear lumen diameter at 1 turn.

IV. 3D printed biomimetic cochleae

	Model descriptors		Definitions	Human variation	Range of the model descriptors investigated
	Matrix resistivity, ρ_{matrix} (kΩcm)	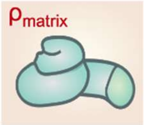	Resistivity of the electro-mimetic bone matrix	0.6 – 26.6	0.2 ± 0.1 – 23 ± 4
Geometric descriptors	Basal lumen diameter, BL_d (mm)	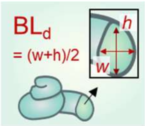	The average of the width (w) and the height (h) of the cross-sectional area of the entrance of the cochlear lumen	1.6 – 2.6	2.00 ± 0.02 – 2.50 ± 0.02
Geometric descriptors	Taper ratio, Tr_a	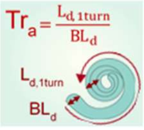	The ratio of the lumen diameter at 1 turn ($L_{d,1turn}$) to the basal lumen diameter (BL_d)	0.3 – 1.0	0.60 ± 0.01 – 0.90 ± 0.06
Geometric descriptors	Cochlear width, W_c (mm)	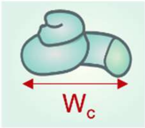	The largest distance from the entrance to the contralateral wall	7.4 – 11.9	7.30 ± 0.04 – 12.7 ± 0.3
Geometric descriptors	Cochlear height, h_c (mm)	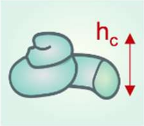	The largest distance between the apical-most point and the basal-most point of the cochlea	3.3 – 6.2	3.5 ± 0.1 – 5.0 ± 0.1

Supplementary Table 1| Definitions and the ranges of the model descriptors investigated in this study in comparison with the reported variation in human cochleae[22–25].

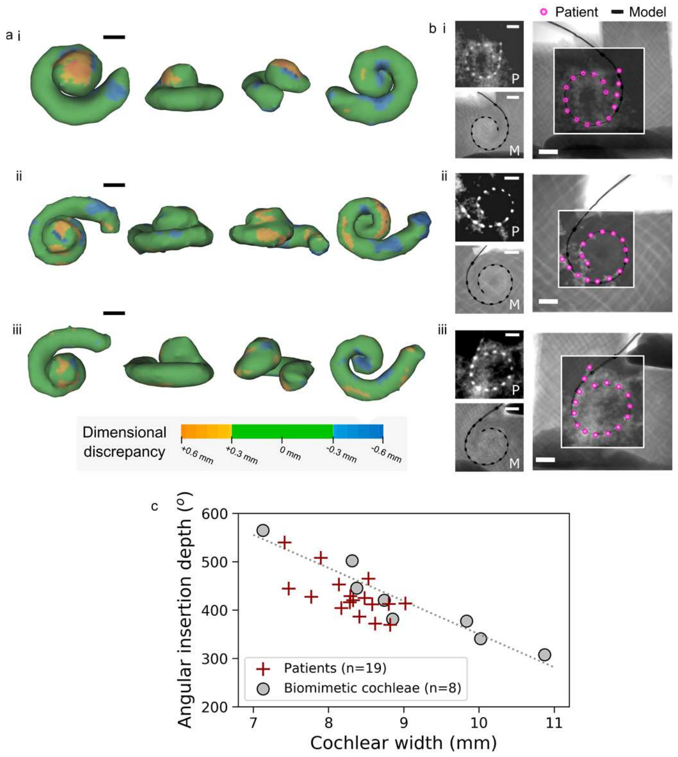

Supplementary Fig.7| Biomimetic cochleae capturing the electrode positions in patient cochlea.

a, Comparison of the dimensional discrepancies between patients' cochlear CT and the lumen of the 3D printed biomimetic cochleae that have similar geometrical descriptors. The dimensional discrepancy is encoded with color with a defined tolerance of ± 0.3 mm, which is the mean pixel size of the patients' CT scans, using AutoDesk Recap Photo. Their geometrical descriptors are as follows: i) BL_d ~ 2.2 mm, Tr_a ~ 0.81, W_c ~ 8.9 mm and h_c ~ 4.6 mm, ii) BL_d ~ 2.0mm, Tr_a ~ 0.9, W_c ~ 8.7 mm and h_c ~ 4.7 mm and iii) BL_d ~ 2.0 mm, Tr_a ~ 0.87, W_c ~ 8.4 mm and h_c ~ 4.8 mm. Scale bar = 2 mm.

b, The electrode positions of the patients' cochleae (P) showed in (a) and their corresponding 3D printed models (M). The images on the upper left, lower left and right show the clinical plain x-ray image of the positions of the CI^{SlimJ} electrodes in the patient's cochlea, the µ-CT image of the positions of the CI^{SlimJ} electrodes in the cochlear lumen of the 3D printed cochlea, and their overlap image to show their similarity in the electrode positions and the angular insertion depth. The angular insertion depths are i) ~420°, ii) ~429° and iii) 380°. Scale bar = 2 mm.

c, The relationship between the angular insertion depth of the CI electrode array and the cochlear width in patients' cochleae (n = 19) and in the 3D printed biomimetic cochleae (n = 8) with different geometric descriptors. The 3D printed biomimetic cochleae approximately display a similar trend to the patients' trend.

V. 3PNN – '3D printing and neural network co-modelling'

Supplementary Note 2| Rationale for using neural network as a modelling approach compared to existing modelling approaches.

Computational models that correctly describe the behaviour of experimental data are beneficial when large amount of experimental data is needed for recognising trends in datasets. Experiments are typically time-consuming and sometimes expensive to perform, thus, computational models can be used as a surrogate model to predict the outcome without the need of additional experiments. Computational models also make determining some physically relevant parameters possible. Therefore, choosing the right modelling approach is of particular importance to reduce the discrepancy between predictions and experimental results.

In this study, we proposed using a multilayer perceptron (MLP) neural network (NN) to model how the EFI depends on the cochlear resistivity, basal lumen diameter, taper ratio, cochlear width and cochlear height. Alternative methods such as the classical parametric regression models and the 'lumped parameter models' were comparatively evaluated for our application.

Parametric regression models often require knowledge of the explicit theoretical functional form that describes the data behaviour. This method is inappropriate for our study because we do not have a theoretical functional form that well describes the voltage distribution from a current source in a heterogeneous medium. On the contrary, artificial neural networks are purely data-driven models. They are known as universal approximators — they have the ability to learn complex and non-linear mappings between the inputs and outputs without the need of knowledge of the explicit model dynamics[26]. Therefore, neural networks usually outperform and achieve a better fit than classical regression models when 1) the explicit functional form of the data behaviour is not known/perfect, 2) non-linearities are involved, and 3) the number of variables is large. These properties are beneficial for modelling our problem.

Several studies have developed 'lumped parameter models' to simulate the *in vivo* EFI profiles[27–29]. These models (Supplementary Fig.8a) simplify the description of the spatially varied electrical resistance of cochleae with a discrete electrically equivalent circuit consisting of a number of parameters, which may have direct physical meanings. To explore the feasibility of using a lumped parameter model to build an analytical model for our application here, we examined whether solutions of parameters are identifiable in backward calculation from EFI profiles. We attempted to infer the parameters of the lumped parameter model from patients' EFI profiles. The lumped parameter model used in these studies was based on the 1st order leaky transmission line tissue model (Supplementary Fig.8a) developed by Vanpoucke et al.[27]. Supplementary Fig.8b shows the solutions of the parameters found from 4 patients' EFI profiles. For each patient EFI, the model was solved 3 times to check if the solutions were converged. We found that several parameters of the lumped parameter model were non-identifiable with our patients' EFI data. In particular, the parameters at the

basal-most and apical-most ends (electrodes 1 and 16) could be inconsistent in different iterations. In addition, the results indicate that the transversal resistance r_T changes abruptly across the electrode array. Previous study from Vanpoucke *et al.* attributed this abrupt dip at e7 – e8 to the current drain through facial nerve[27]. However, in our results, the dip happened at several locations along the electrode array, not just at e7 – e8. This may suggest r_T extrapolated at specific electrode location may not be physically meaningful. Most importantly, the lumped parameter model is not able to reveal the anatomical dependence of EFI.

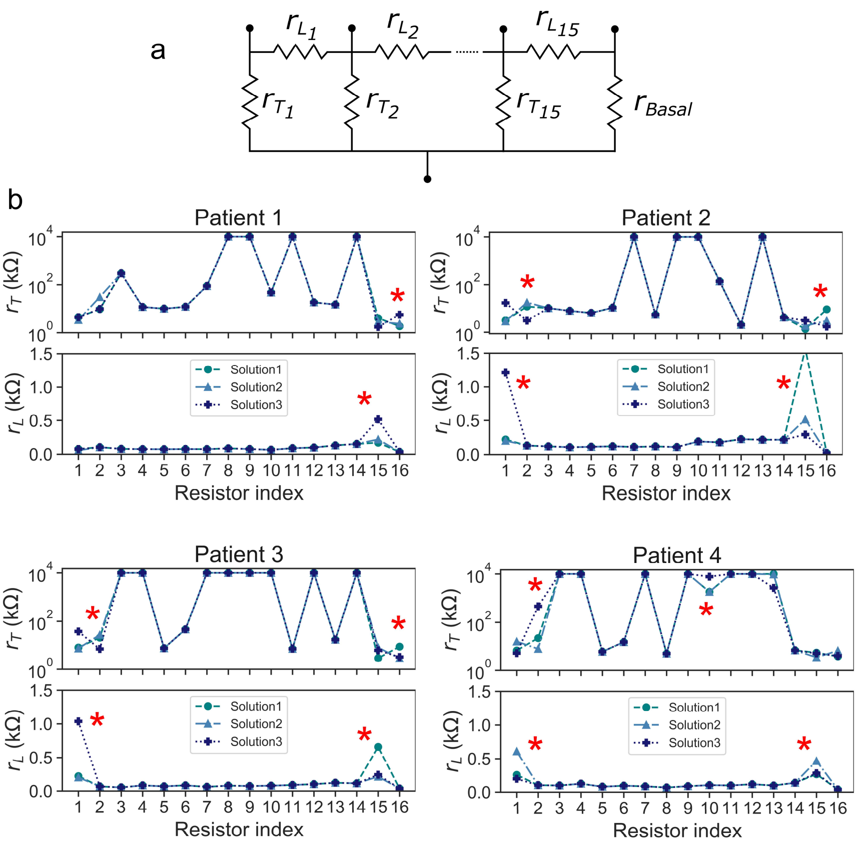

Supplementary Fig.8| Lumped parameter model for modelling volume conduction in cochleae with our patients' data.

- **a,** A 1st order leaky transmission line model proposed by Vanpoucke et al. for modelling the electrical volume conduction within cochleae[27]. Each segment represents a section of an uncoiled cochlea between subsequent electrodes. The longitudinal resistors (r_L) represent the current flow along the cochlear lumen, the transversal resistors (r_T) represent the current flow through the lateral and modiolar bony structures, and the basal resistor (r_{Basal}) represents the resistance between the base of cochlea and the reference electrode.

- **b,** The solutions of the parameters of the lumped parameter model solved by backward calculation of 4 patients' EFI profiles. * denotes the parameter identifiability issue of the model where the solutions were different over 3 iterations.

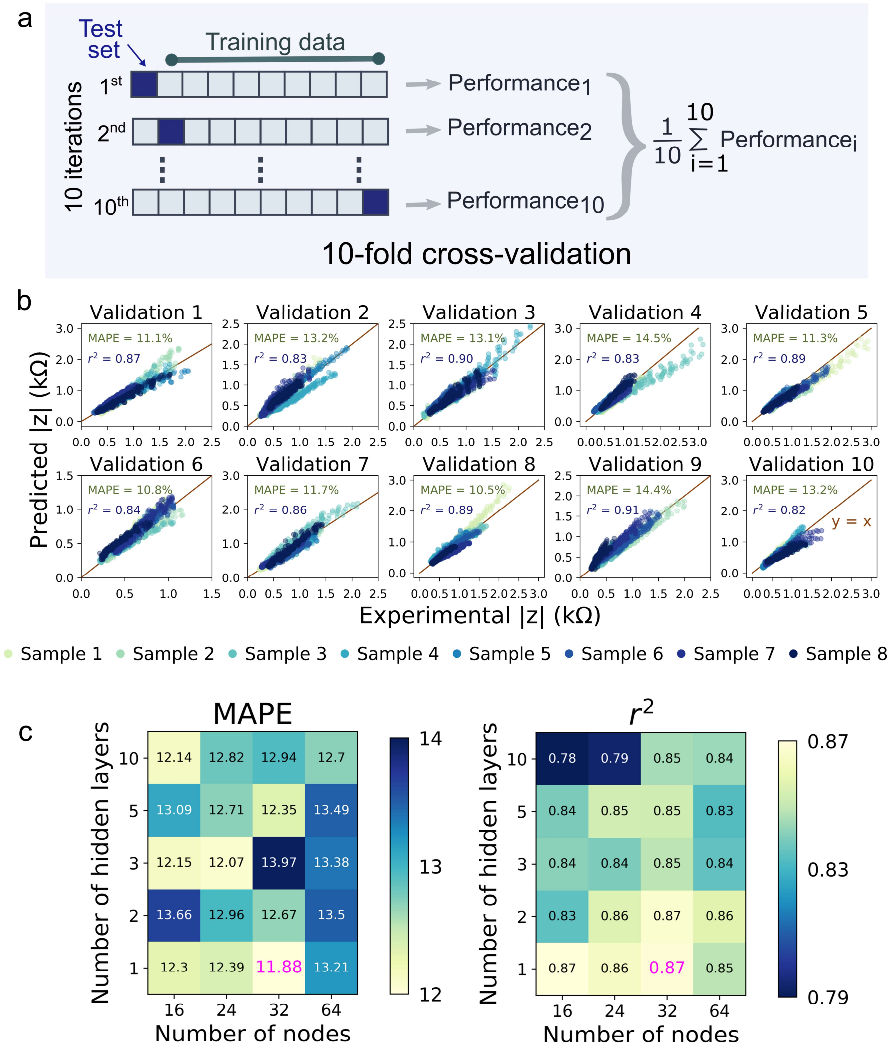

Supplementary Fig.9| Hyperparameter tuning of 3PNN.

a, 10-fold cross-validation, a standard procedure for estimating the performance of neural network models[30], was used to quantify the accuracy of 3PNN. In the 10-fold cross validation, our dataset of 82 EFI profiles, associated with a variety of electroanatomical cochlear features, was randomly split into 10 subsets. One subset was used as an unseen subset (test subset) for prediction while the remaining subsets were used for training.

b, An example showing the comparison between the predictions and the actual experimental off-stimulation EFI data of CI[1J] in a 10-fold cross validation. The inputs of the stimulating and the recording electrode positions are listed in Supplementary Table 2. Each colour represents an unseen sample in an iteration. The deviation of the points from the identity line (shown as red lines) indicates how much the predicted values deviate from the experimental data. The average median absolute percentage error (MAPE) and the average R^2 score were used to quantify the predictive performance of the NN model.

c, A grid search was performed to tune the hyperparameters (number of hidden layers and number of nodes) of the NN model that give the optimal performance of the NN model. We varied the number of hidden layers from 1 to 10 (1, 2, 3, 5, 10) and the number of nodes in each hidden layer from 16 to 64 (16, 24, 32, 64). For each combination of hyperparameters, a 10-fold cross-validation was performed. This figure summarises the average MAPE and the average R^2 score of the model trained with a specific combination of hyperparameters. We found that the performance is optimal when the number of hidden layer = 1 and number of nodes in each hidden layer = 32 (with the smallest average MAPE and the highest average R^2, indicated in purple). Hence, this set of hyperparameters was selected for training the final NN model. In summary, the input layers consist of 7 parameters – basal lumen diameter, taper ratio, cochlear height, cochlear width, matrix resistivity, an array of the stimulating electrode positions and an array of the recording electrode positions; one hidden layer with 32 nodes is used; the output of the NN model is a transimpedance matrix (known as EFI for Advanced Bionics® implants, or transimpedance matrix for Cochlear Corporation® implants), of which the dimension equals to the product of the dimension of the recording position array and the dimension of the stimulating position array.

CI electrode array	Electrode spacing (mm)	Electrode positions along the CI (mm)
Advanced Bionics® HiFocus™ SlimJ electrode (CI^{SlimJ})	1.3	[3, 4.3, 5.6,…., 17.3, 18.6]
Advanced Bionics® HiFocus™ 1J electrode (CI^{1J})	1.1	[2, 3.1, 4.2,…., 17.4, 18.5]
Cochlear ™ Nucleus® slim straight electrode (CI622)	0.9	[3.85, 4.75, 5.65,…., 17.35, 18.25]
Cochlear ™ Nucleus® slim straight electrode (CI522)	0.9	[3.85, 4.75, 5.65,…., 17.35, 18.25]

Supplementary Table 2| Input values of the stimulating and the recording electrode positions of different electrode types used in 3PNN.

We assume that the stimulating and the recording electrode positions follow the CI specifications in all predictions[21,31,32]. Note that CI622 and CI522 have the same electrode spacing and the electrode positions. As CI622 and CI522 do not have a linear electrode spacing (ranges from 0.85 to 0.95 mm), the average electrode spacing of CI522 and CI622 (0.9 mm) is used.

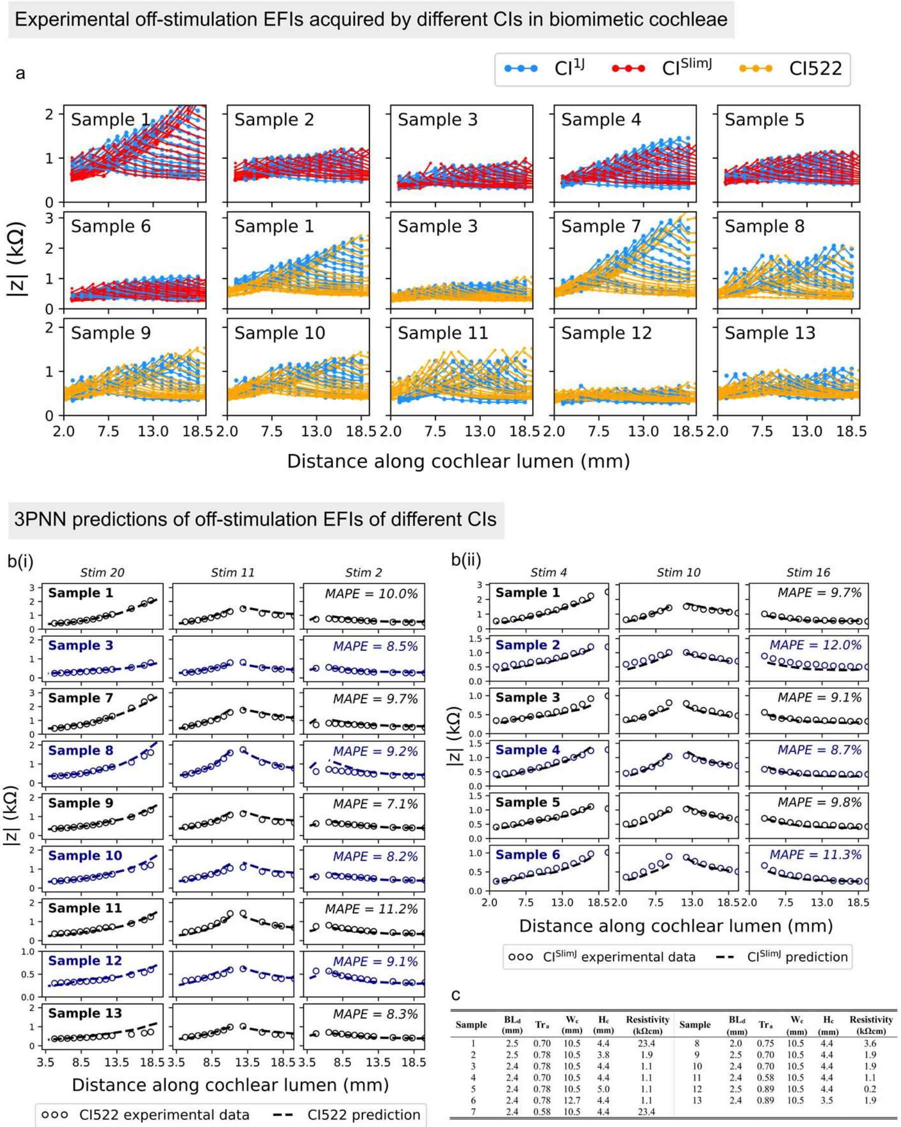

Sample	BL_d (mm)	Tr_a	W_c (mm)	H_c (mm)	Resistivity (kΩcm)	Sample	BL_d (mm)	Tr_a	W_c (mm)	H_c (mm)	Resistivity (kΩcm)
1	2.5	0.70	10.5	4.4	23.4	8	2.0	0.75	10.5	4.4	3.6
2	2.5	0.78	10.5	3.8	1.9	9	2.5	0.70	10.5	4.4	1.9
3	2.4	0.78	10.5	4.4	1.1	10	2.4	0.70	10.5	4.4	1.9
4	2.4	0.70	10.5	4.4	1.1	11	2.4	0.58	10.5	4.4	1.1
5	2.4	0.78	10.5	5.0	1.1	12	2.5	0.89	10.5	4.4	0.2
6	2.4	0.78	12.7	4.4	1.1	13	2.4	0.89	10.5	3.5	1.9
7	2.4	0.58	10.5	4.4	23.4						

Supplementary Fig.10| Applicability of 3PNN on different electrode types.

a, Experimental off-stimulation EFIs or transimpedance matrices acquired by either CI^{1J}, CI^{SlimJ} or CI522 in same biomimetic cochlea samples. The similarity between the CI^{1J} and CI^{SlimJ} EFIs were showed by 6 samples, and the similarity between CI^{1J} EFIs and the CI522 transimpedance matrices was demonstrated by 9 samples. The specifications of the 3D printed samples can be found in (c). The results show that the overall shape and the trend of EFIs (or transimpedance matrices) are weakly dependent of the electrode design. This gives confidence that 3PNN, which is trained by CI^{1J}, can be broadly implemented for different CI electrode types.

b, Accuracy of 3PNN in predicting (i) CI522 transimpedance matrices and (ii) CI^{SlimJ} EFIs. MAPE is used as a measure of the discrepancy between the predicted and the experimental data measured in the biomimetic cochleae. The inputs of the electrode positions defined in 3PNN can be found in Supplementary Table 2.

c, Specifications of the samples tested here.

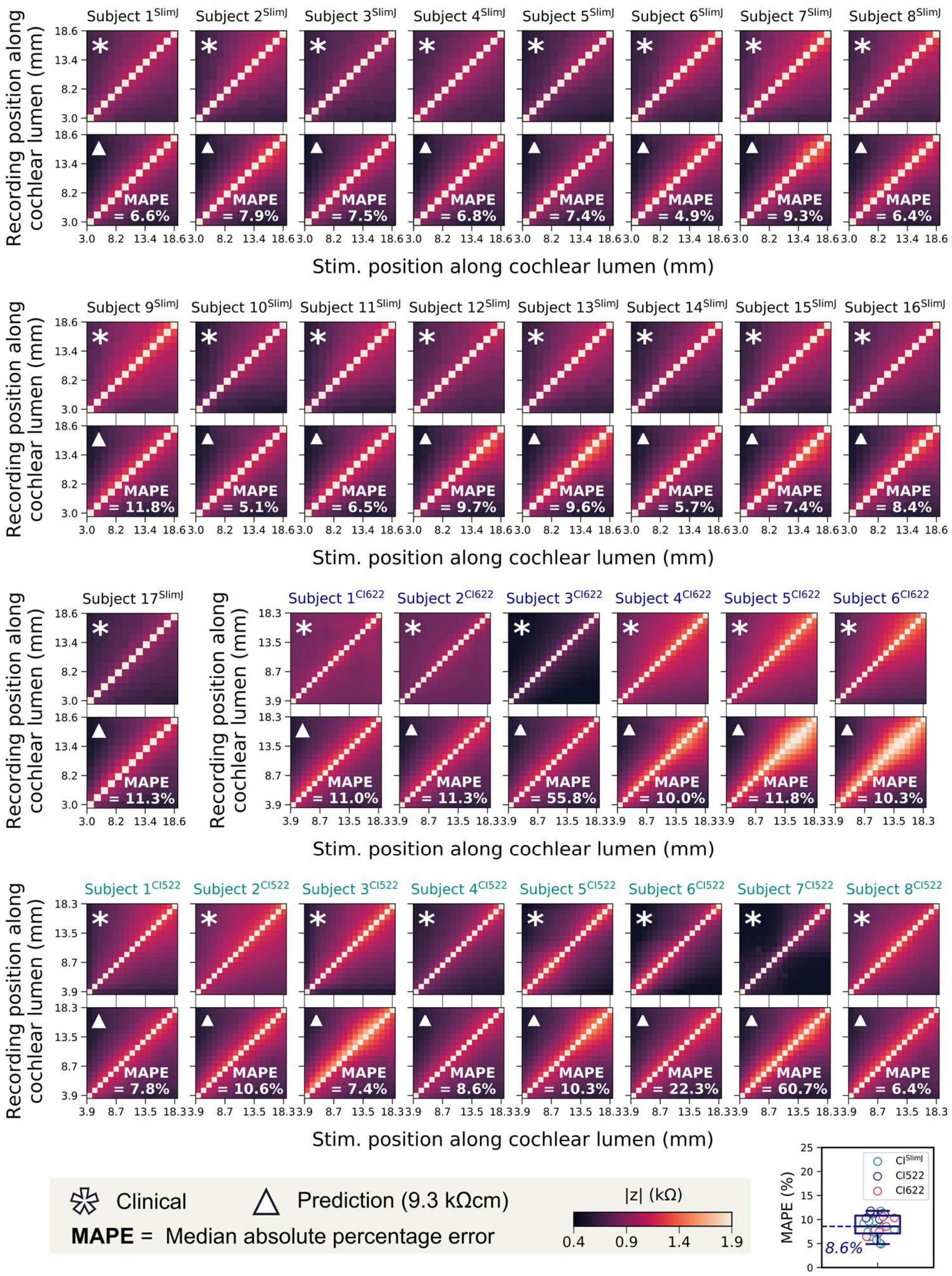

Supplementary Fig.11| Validation of forward-3PNN.

Comparison of the patients' clinical EFIs and the off-stimulation **EFIs** predicted by forward-3PNN **using the four geometric descriptors** measured from patients' cochlear CTs. In all predictions, the matrix resistivity inputs were defaulted as the mean reported resistivity of live human skulls (9.3 kΩcm, Supplementary Fig.2). Three CI electrode types were examined here; they are CI^{SlimJ} (n = 17), CI622 (n = 6) and CI522 (n = 8). The inputs of the stimulating and the recording electrode positions were assumed to follow the suggested insertion depth in their implant specification (Supplementary Table 2). MAPE between the patient's and the predicted off-stimulation EFIs is used as a measure of the prediction error (summarised in the boxplot at the lower right). Overall, 3PNN exhibits a median MAPE of 8.6%. The line in the box of the boxplot shows the median, with the box denoting the interquartile range and the whiskers denoting the ± 1.5 of the interquartile range.

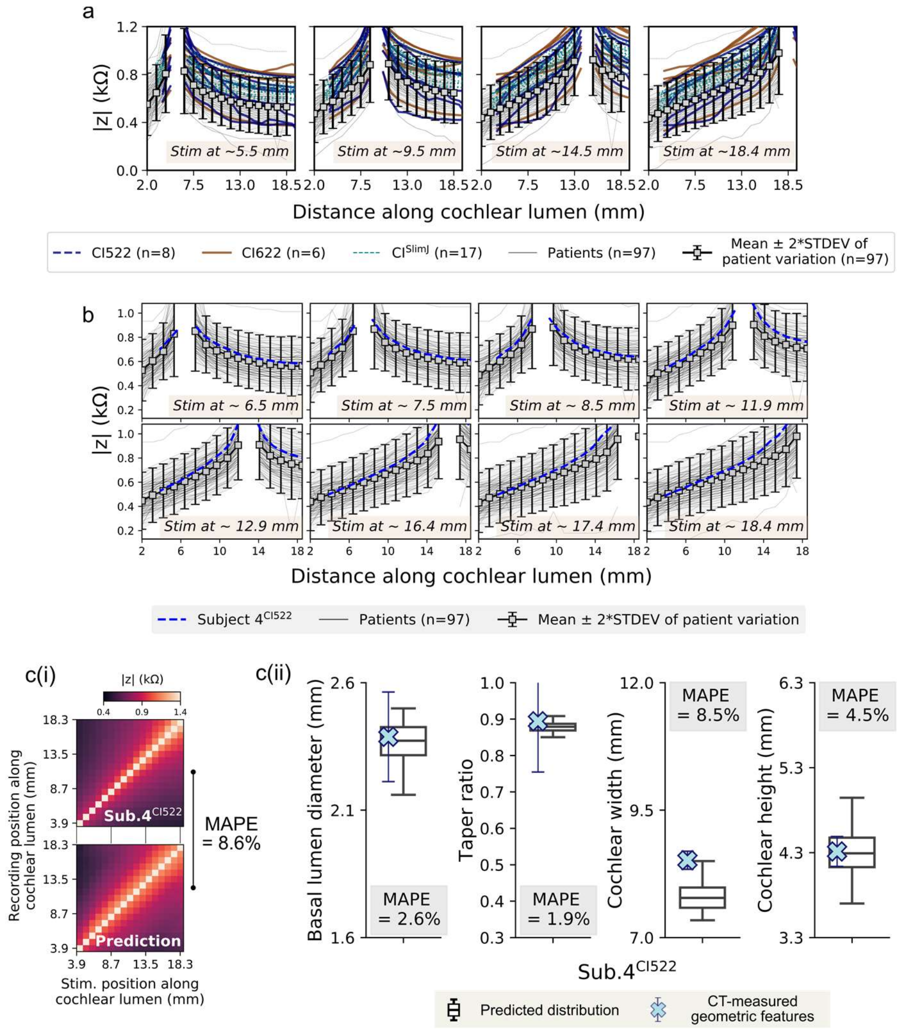

Supplementary Fig.12| Predictive performance of 3PNN against the EFI profiles within patient population.

- **a,** The clinical EFIs (n = 31) used in the validation of 3PNN to represent the EFI variation in patient population (n = 97). EFIs induced by the stimulations at ~ 5.5 mm, ~ 9.5 mm, ~ 14.5 mm and ~ 18.4 mm were shown. Note that the patient variation can be mostly captured by two standard deviations, as shown in (b).

- **b,** The mean of the patients' EFI profiles (or transimpedance matrix profiles) (n = 97). Clinical profile of subject 4^{CI522} is used to represent the patient population mean.

- **c,** Performance of **(i)** forward-3PNN and **(ii)** inverse-3PNN on subject 4^{CI522}. Error bars of the patients' CT-measured geometric features are approximated as half pixel size of the CT scans. Both forward-3PNN and inverse-3PNN exhibit a good accuracy, with < 9% MAPE. The box plots in (ii) represent the predicted posterior distribution by randomly drawing 1,000 samples from the distribution (n = 1,000). The line in the box shows the median, with the box denoting the interquartile range and the whiskers denoting the ± 1.5 of the interquartile range.

	Subjects	MAPE between the 3PNN-predicted EFI and the actual subject's EFI (%)	MAPE between the mean patient EFI and the actual subject's EFI (%)
CI^{SlimJ}	Subject 1^{SlimJ}	6.6	21.4
	Subject 2^{SlimJ}	7.9	18.9
	Subject 3^{SlimJ}	7.5	13.3
	Subject 4^{SlimJ}	6.8	19.0
	Subject 5^{SlimJ}	7.4	8.1
	Subject 6^{SlimJ}	4.9	17.0
	Subject 7^{SlimJ}	9.3	25.2
	Subject 8^{SlimJ}	6.4	23.5
	Subject 9^{SlimJ}	11.8	29.7
	Subject 10^{SlimJ}	5.1	10.8
	Subject 11^{SlimJ}	6.5	17.2
	Subject 12^{SlimJ}	9.7	20.3
	Subject 13^{SlimJ}	9.6	18.9
	Subject 14^{SlimJ}	5.7	11.0
	Subject 15^{SlimJ}	7.4	18.9
	Subject 16^{SlimJ}	8.4	21.4
	Subject 17^{SlimJ}	11.3	11.8
CI622	Subject 1^{CI622}	11.0	29.0
	Subject 2^{CI622}	11.3	23.0
	Subject 3^{CI622}	55.8	7.0
	Subject 4^{CI622}	10.0	38.2
	Subject 5^{CI622}	11.8	40.6
	Subject 6^{CI622}	10.3	41.4
CI522	Subject 1^{CI522}	7.8	36.2
	Subject 2^{CI522}	10.6	39.2
	Subject 3^{CI522}	7.4	37.5
	Subject 4^{CI522}	8.6	28.2
	Subject 5^{CI522}	10.3	24.5
	Subject 6^{CI522}	22.3	16.2
	Subject 7^{CI522}	60.7	5.0
	Subject 8^{CI522}	6.4	29.9
	**Median**	**8.6**	**21.4**
	**Interquartile range (IQR)**	**7.1 – 10.8**	**16.6 – 29.3**

Supplementary Table 3| 3PNN performance compared with the MAPE obtained from the population mean EFI.

Table showing first column, the MAPE values between the 3PNN prediction (using known patient geometric factors) and the corresponding patient' off-stimulation EFI; and second column, the MAPE values calculated from the patient mean (n = 97) and each patient's off-stimulation EFIs. Since different CI electrode arrays have different electrode positions, comparisons with the patient mean EFI were made by comparing the transimpedance values associated with similar electrode positions (± 0.2 mm).

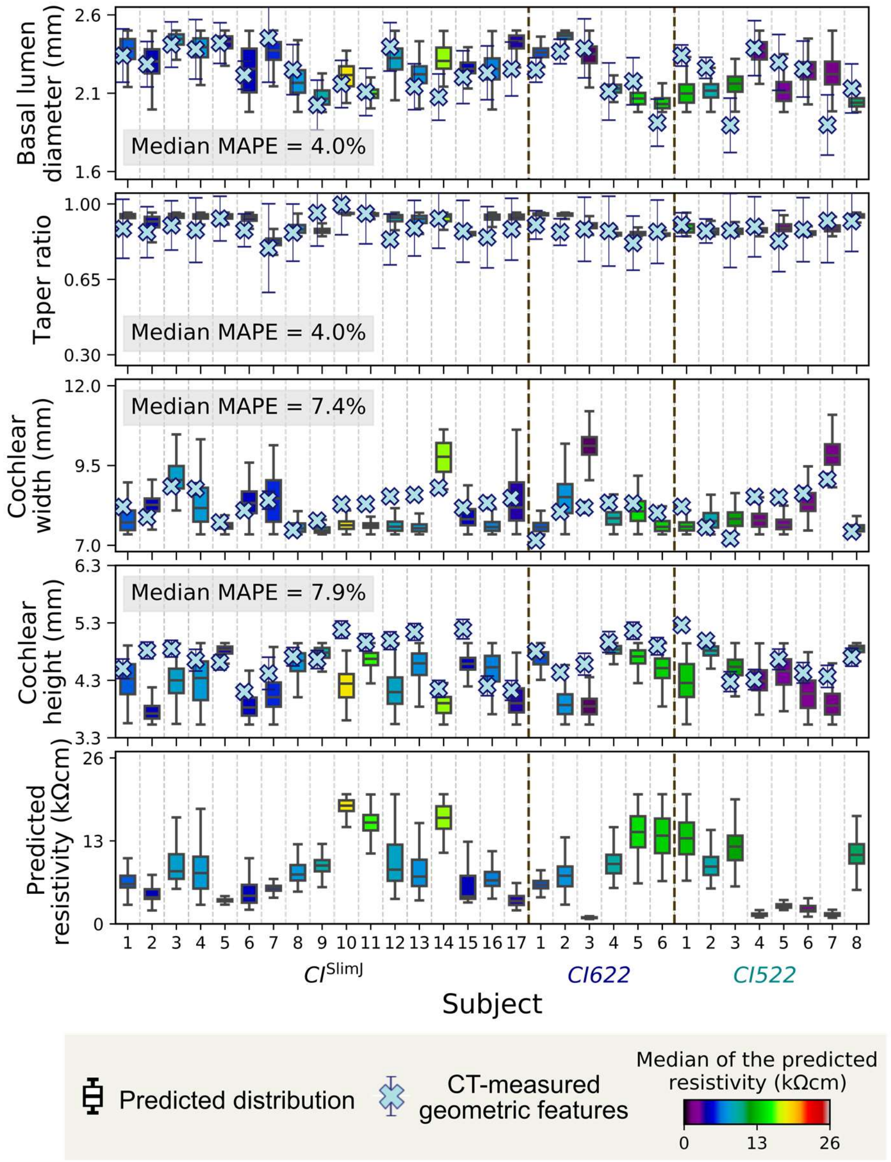

Supplementary Fig.13| Validation of inverse-3PNN.

Comparison of the patients' geometric descriptors measured from their clinical cochlear CT scans and the predicted distributions of the geometric descriptors. The predicted posterior distributions were found using inverse-3PNN and were approximated by sampling 1,000 points in each prediction. Detailed values of the MAPE threshold used in each prediction can be found in Supplementary Table 8. The line in the box shows the median of the predicted values of the descriptor, with the box denoting the interquartile range and the whiskers denoting the ± 1.5 of the interquartile range. Three CI electrode types were examined here; they are CI^{SlimJ} (n = 17), CI622 (n = 6) and CI522 (n = 8). MAPE is used to quantify the error between a patient's CT-measured geometric feature and the predicted distribution,. Median MAPE is used as a measure of the overall error in the predictions of each geometric descriptor. The colours of the boxplots indicate the median of the predicted resistivity. Error bars of the patients' CT-measured geometric features are approximated as half pixel size of the CT scans. The predictions of the patient-specific cochlear resistivity could not be validated due to no reported method of measuring cochlear resistivity of live human.

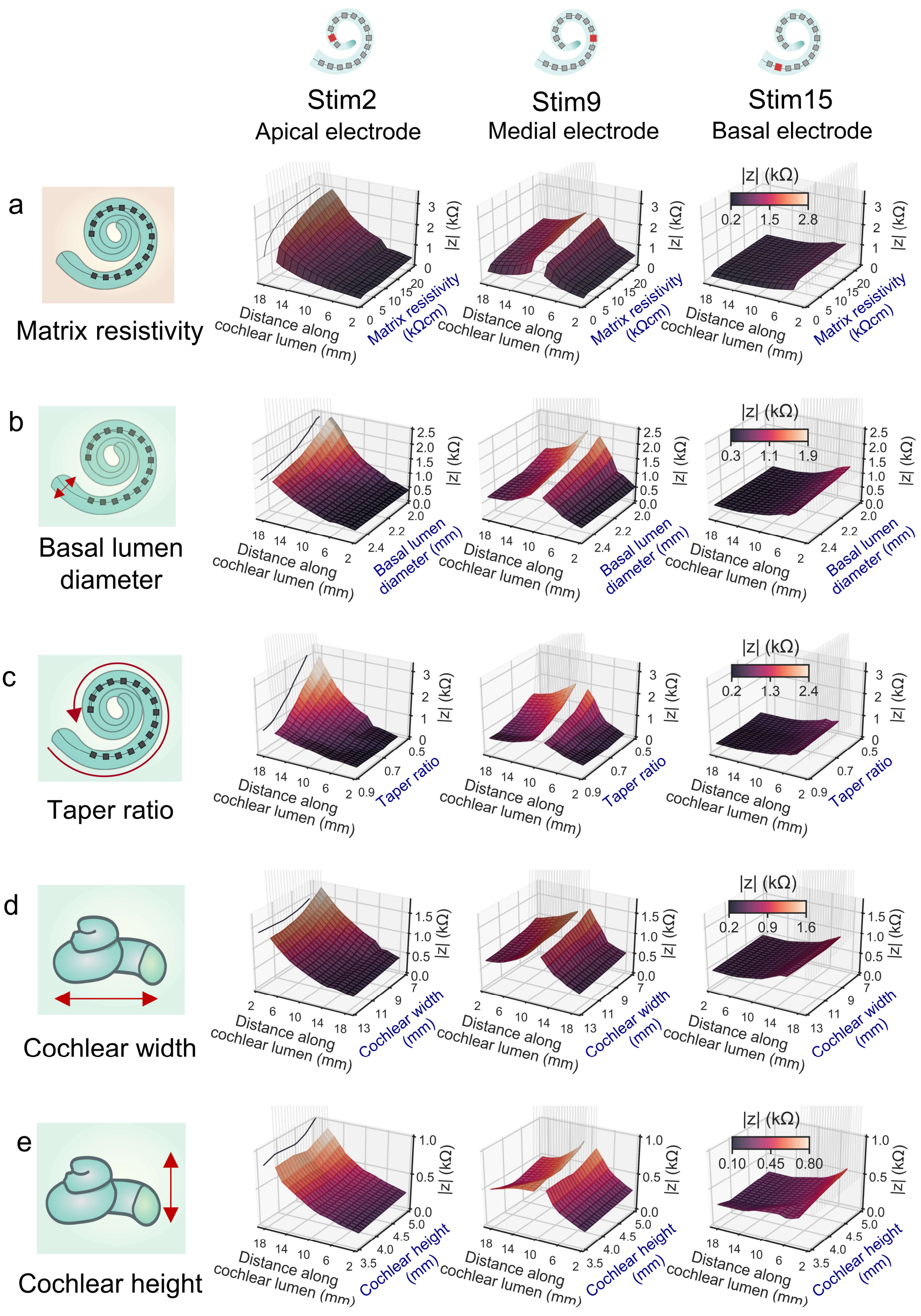

Supplementary Fig.14| Example showing the trend of the predicted EFI profiles along the 5 model descriptors.

The trend of EFIs along **(a)** matrix resistivity, **(b)** basal lumen diameter, **(c)** taper ratio, **(d)** cochlear width and **(e)** cochlear height. In each graph, only one descriptor varies, while other descriptors are held constant. For visualization purpose, only the transimpedance magnitude $|z|$ induced by the stimulations at the apical electrode (electrode 2), the medial electrode (electrode 9) and the basal electrode (electrode15) were shown here.

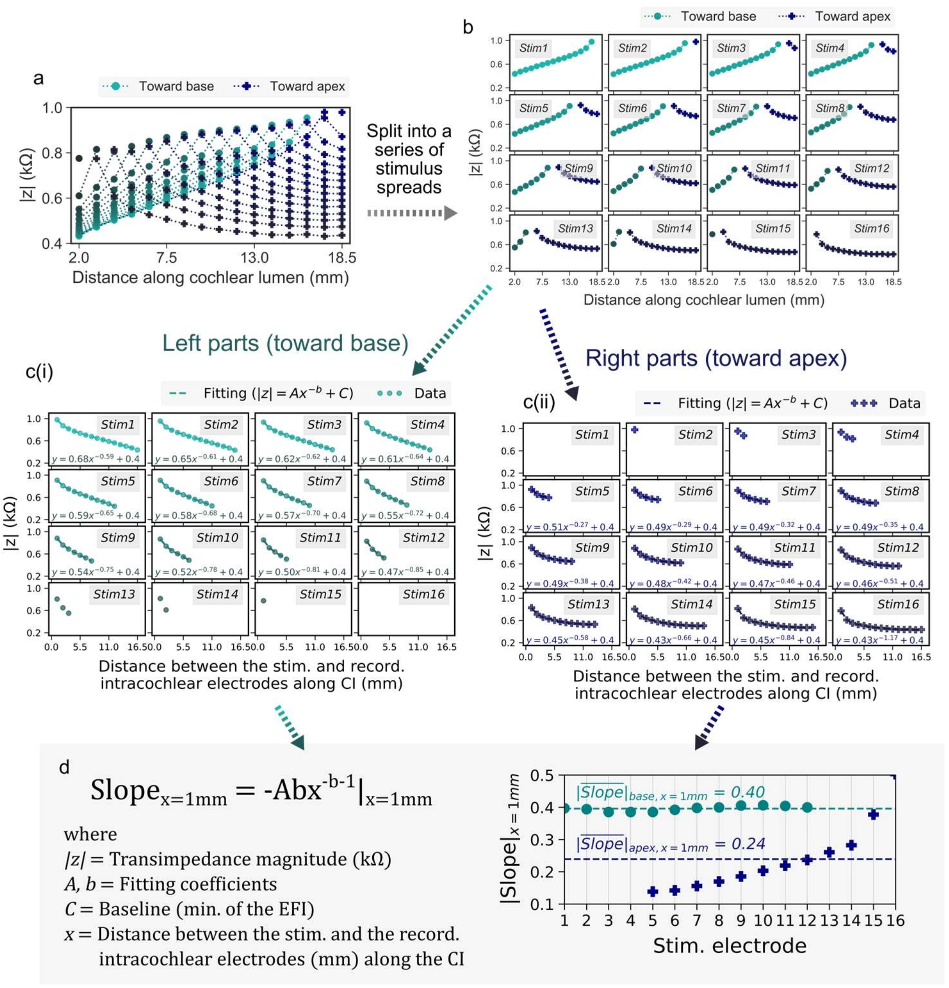

Supplementary Fig.15| Example of the power-law fitting analysis of EFIs.

a, An EFI profile with colours indicating the spreads toward the apex and the base.

b, The profile was split into a series of stimulus spreads. Each stimulus spread is induced by a stimulation of a CI electrode.

c, Each stimulus spread is split into two parts – **(i)** spread toward the base (left) and **(ii)** spread toward the apex of a cochlea (right). The x-axis was converted to the distance between the stimulating and the recording intracochlear electrodes along the CI. Fitting of a power law equation ($|z| = Ax^{-b} + C$) was performed for each directional stimulus spread that has a minimum of 4 points.

d, The slope of each stimulus spread was computed by calculating the derivative of the fitting equation with respect to x. The graph summarises the magnitudes of $|\mathrm{Slope}|_{x=1mm}$ of the stimulus spreads toward the apex and toward the base associated with different stimulating electrodes. The mean of the spreads toward the apex ($\overline{|\mathrm{Slope}|}_{apex,\ x=1mm}$) and the mean of the spreads toward the base ($\overline{|\mathrm{Slope}|}_{base,\ x=1mm}$) are used to quantify the sharpness of voltage drop toward the base and the sharpness toward the apex in an EFI.

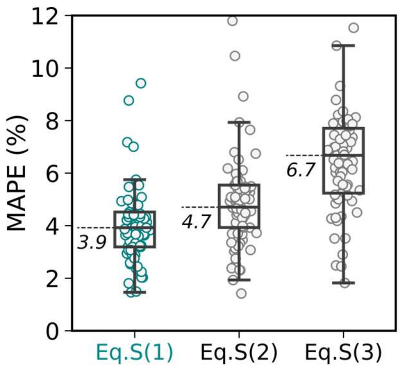

Supplementary Fig.16| Goodness-of-fit test to evaluate the choice of the fitting forms.

Three potential functional forms that are able to represent the decay features and the baseline features of EFIs are evaluated here, where $|z|$ is the transimpedance magnitude, x is the distance between the stimulating and the recording intracochlear electrodes along the CI, A and b are fitting coefficients, and C is the baseline constant of the EFI.

$$|z| = Ax^{-b} + C \qquad \text{-- Supplementary Equation (1)}$$

$$|z| = \frac{A}{x} + C \qquad \text{-- Supplementary Equation (2)}$$

$$|z| = Ae^{-x} + C \qquad \text{-- Supplementary Equation (3)}$$

To examine how well they fit the clinical EFIs, the equations were fit to clinical EFIs of different electrode types ($n = 75$), following the method described in Supplementary Fig.15a – c. MAPE between the clinical data and the expected values from the fitting is used as the goodness-of-fit score. The line in the box shows the median, with the box denoting the interquartile range and the whiskers denoting the ± 1.5 of the interquartile range. From the graph, we found that Supplementary Equation (1) has the best goodness-of-fit score (lowest MAPE). Therefore, Supplementary Equation (1) is used in the fitting analysis in our study.

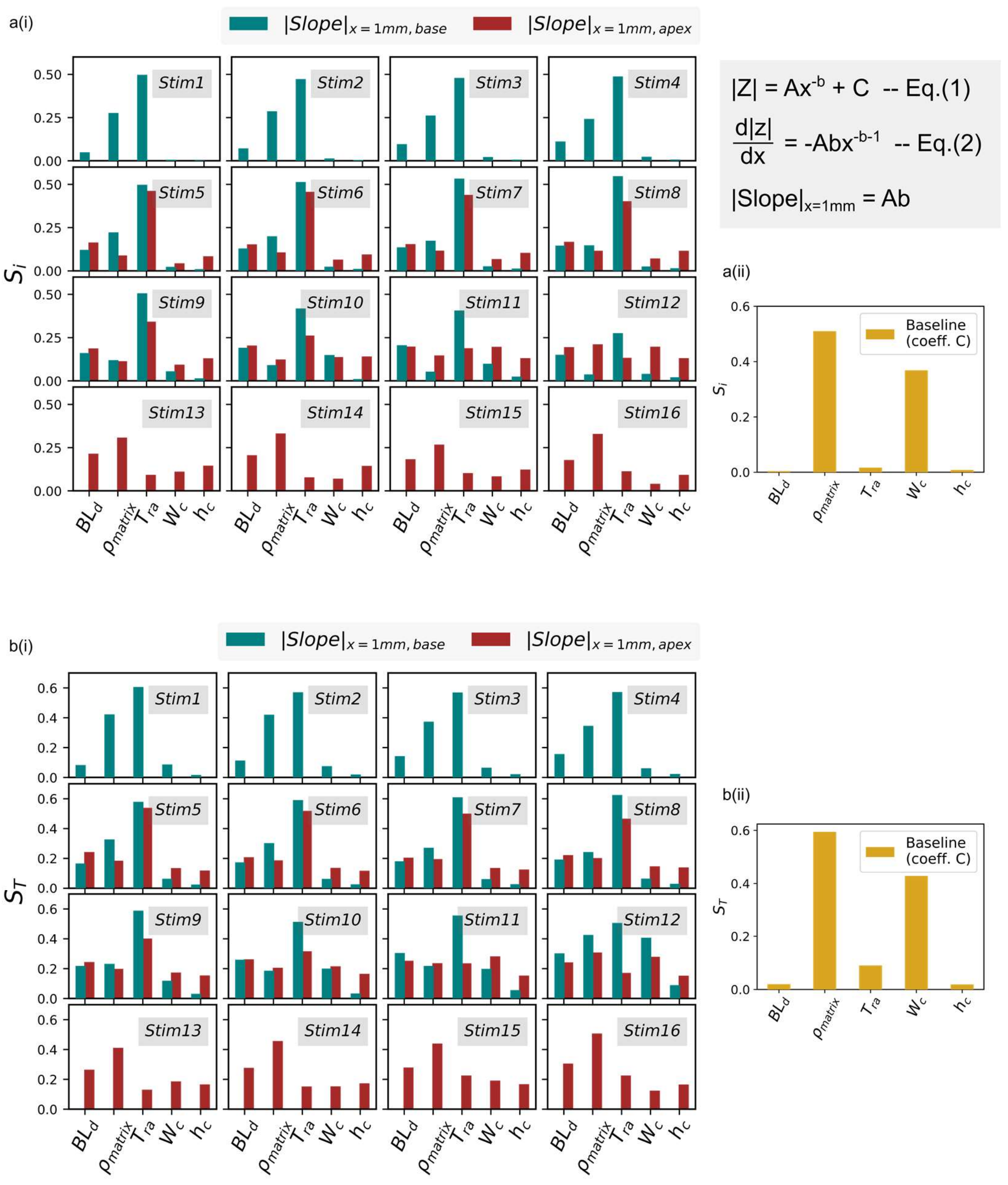

Supplementary Fig.17| First-order and total-order Sobol sensitivity indices of the $|Slope|_{x=1mm}$ and baseline coefficient for different model descriptors.

Global sensitivity analysis was performed using Sobol's method[33]. (a) First-order, S_i, and (b) total-order, S_T, sensitivity indices obtained from Sobol sensitivity analysis on (i) the coefficient product *Ab* ($|slope|_{x=1m}$) toward the cochlear apex and the base associated with different stimulating electrodes, and (ii) the coefficient *C* (the baseline coefficient) associated with Eq.1 and Eq.2 in the main text for different model descriptors. BL_d = basal lumen diameter, ρ_{matrix} = matrix resistivity, Tr_a = taper ratio, W_c = cochlear width and h_c = cochlear height.

The first-order indices reflect the independent importance of each parameter, while the total-order indices reflect the total contribution of a parameter and interaction with other parameters. A larger value of the sensitivity index implies the greater importance of the parameter. The sum of the first order indices, S_i, should be less than or equal to 1.

a) S_{ij} of $|Slope|_{x=1mm}$ (coeff. Ab) toward the base

	Stimulating electrode											
	**1**	**2**	**3**	**4**	**5**	**6**	**7**	**8**	**9**	**10**	**11**	**12**
(BL_d , ρ_{matrix})	0.01	0.01	0.02	0.01	0.01	0.01	0.01	0.01	0.01	0.01	0.00	0.02
(BL_d , Tr_a)	0.01	0.01	0.01	0.01	0.01	0.01	0.01	0.02	0.02	0.02	0.02	0.02
(BL_d , W_c)	0.00	0.00	0.00	0.00	0.00	0.00	0.00	0.00	0.00	0.01	0.01	0.02
(BL_d , h_c)	0.00	0.00	0.00	0.00	0.00	0.00	0.00	0.00	0.00	0.00	0.00	0.01
(ρ_{matrix} , Tr_a)	0.07	0.06	0.05	0.05	0.05	0.04	0.04	0.03	0.03	0.04	0.05	0.03
(ρ_{matrix} , W_c)	0.05	0.03	0.02	0.02	0.02	0.02	0.02	0.02	0.03	0.01	0.03	0.16
(ρ_{matrix} , h_c)	0.00	0.00	0.00	0.00	0.00	0.00	0.00	0.00	0.00	0.00	0.01	0.01
(Tr_a , W_c)	0.00	0.00	0.00	0.00	0.00	0.00	0.00	0.00	0.00	0.00	0.00	0.02
(Tr_a , h_c)	0.00	0.00	0.01	0.01	0.01	0.00	0.00	0.00	0.00	0.00	0.00	0.01
(W_c , h_c)	0.00	0.00	0.00	0.00	0.00	0.00	0.00	0.00	0.00	0.00	0.00	0.01

b) S_{ij} of $|Slope|_{x=1mm}$ (coeff. Ab) toward the apex

	Stimulating electrode											
	**5**	**6**	**7**	**8**	**9**	**10**	**11**	**12**	**13**	**14**	**15**	**16**
(BL_d , ρ_{matrix})	0.02	0.02	0.02	0.01	0.02	0.02	0.02	0.02	0.02	0.03	0.04	0.05
(BL_d , Tr_a)	0.01	0.01	0.01	0.01	0.01	0.01	0.01	0.00	0.00	0.01	0.02	0.02
(BL_d , W_c)	0.01	0.01	0.00	0.00	0.00	0.00	0.00	0.00	0.00	0.00	0.00	0.00
(BL_d , h_c)	0.00	0.00	0.00	0.00	0.00	0.00	0.00	0.00	0.00	0.01	0.01	0.01
(ρ_{matrix} , Tr_a)	0.01	0.01	0.01	0.00	0.01	0.01	0.01	0.01	0.01	0.02	0.04	0.02
(ρ_{matrix} , W_c)	0.02	0.03	0.03	0.03	0.03	0.04	0.05	0.05	0.05	0.04	0.04	0.02
(ρ_{matrix} , h_c)	0.00	0.01	0.00	0.00	0.00	0.01	0.01	0.01	0.01	0.01	0.01	0.02
(Tr_a , W_c)	0.01	0.01	0.01	0.01	0.01	0.01	0.01	0.01	0.00	0.01	0.03	0.02
(Tr_a , h_c)	0.00	0.00	0.00	0.00	0.00	0.00	0.00	0.00	0.00	0.00	0.01	0.01
(W_c , h_c)	0.00	0.00	0.00	0.00	0.00	0.00	0.01	0.01	0.00	0.00	0.00	0.00

c) S_{ij} of baseline (coeff. c)

	Baseline
(BL_d , ρ_{matrix})	0.00
(BL_d , Tr_a)	0.00
(BL_d , W_c)	0.00
(BL_d , h_c)	0.00
(ρ_{matrix} , Tr_a)	0.03
(ρ_{matrix} , W_c)	0.02
(ρ_{matrix} , h_c)	0.00
(Tr_a , W_c)	0.01
(Tr_a , h_c)	0.00
(W_c , h_c)	0.00

Supplementary Table 4| Second-order Sobol sensitivity indices of the $|Slope|_{x=1mm}$ and baseline coefficient.

Second-order indices, S_{ij}, of the coefficient product *Ab* ($|slope|_{x=1mm}$) toward **(a)** the cochlear base and **(b)** the apex associated with different stimulating electrodes, and **(c)** the coefficient *C* (the baseline coefficient) associated with Eq.1 and Eq.2 in the main text for any two model descriptors. BL_d = basal lumen diameter, ρ_{matrix} = matrix resistivity, Tr_a = taper ratio, W_c = cochlear width and h_c = cochlear height. The small values of the second-order indices indicate the variance caused by the interaction of any two model descriptors is less significant.

a) S_i of EFI for basal lumen diameter (BL_d)

Recording electrodes		Stimulating electrodes 1	2	3	4	5	6	7	8	9	10	11	12	13	14	15	16
	1	0.00	0.00	0.00	0.00	0.00	0.00	0.00	0.00	0.01	0.01	0.01	0.02	0.05	0.10	0.15	0.16
	2	0.00	0.00	0.01	0.01	0.01	0.01	0.01	0.01	0.01	0.01	0.02	0.03	0.06	0.11	0.12	0.07
	3	0.01	0.01	0.01	0.01	0.01	0.01	0.01	0.02	0.02	0.04	0.05	0.07	0.10	0.12	0.08	0.06
	4	0.01	0.01	0.01	0.01	0.02	0.02	0.02	0.03	0.04	0.05	0.08	0.12	0.14	0.10	0.07	0.05
	5	0.01	0.01	0.02	0.02	0.02	0.03	0.03	0.04	0.06	0.09	0.14	0.15	0.12	0.09	0.05	0.03
	6	0.01	0.02	0.02	0.03	0.03	0.04	0.04	0.06	0.10	0.14	0.15	0.12	0.09	0.05	0.02	0.01
	7	0.02	0.02	0.02	0.03	0.04	0.05	0.07	0.09	0.12	0.13	0.12	0.09	0.06	0.03	0.01	0.01
	8	0.03	0.03	0.04	0.04	0.05	0.07	0.09	0.11	0.11	0.10	0.08	0.06	0.03	0.01	0.01	0.01
	9	0.03	0.04	0.05	0.05	0.07	0.09	0.10	0.09	0.09	0.08	0.06	0.03	0.01	0.01	0.00	0.01
	10	0.03	0.04	0.05	0.07	0.08	0.09	0.08	0.08	0.07	0.06	0.04	0.02	0.01	0.00	0.00	0.00
	11	0.04	0.05	0.06	0.08	0.08	0.07	0.08	0.07	0.06	0.05	0.02	0.01	0.00	0.00	0.00	0.00
	12	0.04	0.05	0.07	0.08	0.07	0.07	0.07	0.06	0.05	0.03	0.01	0.01	0.00	0.00	0.00	0.00
	13	0.04	0.06	0.07	0.06	0.07	0.07	0.06	0.05	0.03	0.02	0.01	0.01	0.00	0.00	0.00	0.00
	14	0.05	0.06	0.05	0.06	0.06	0.06	0.05	0.04	0.03	0.02	0.01	0.01	0.00	0.00	0.00	0.00
	15	0.05	0.04	0.05	0.05	0.05	0.05	0.04	0.03	0.02	0.01	0.01	0.00	0.00	0.00	0.00	0.00
	16	0.03	0.04	0.04	0.04	0.04	0.03	0.03	0.02	0.02	0.01	0.01	0.00	0.00	0.00	0.00	0.00

b) S_i of EFI for matrix resistivity (ρ_{matrix})

Recording electrodes		Stimulating electrodes 1	2	3	4	5	6	7	8	9	10	11	12	13	14	15	16
	1	0.51	0.51	0.51	0.52	0.52	0.53	0.53	0.53	0.54	0.57	0.60	0.62	0.65	0.63	0.57	0.51
	2	0.51	0.51	0.52	0.53	0.53	0.53	0.53	0.54	0.56	0.57	0.60	0.63	0.63	0.60	0.61	0.61
	3	0.52	0.54	0.54	0.54	0.53	0.52	0.53	0.52	0.52	0.52	0.53	0.54	0.55	0.56	0.55	0.56
	4	0.47	0.50	0.50	0.49	0.48	0.48	0.48	0.46	0.44	0.44	0.45	0.49	0.55	0.54	0.56	0.58
	5	0.41	0.43	0.43	0.42	0.43	0.43	0.41	0.40	0.38	0.37	0.42	0.49	0.50	0.54	0.58	0.61
	6	0.39	0.40	0.40	0.41	0.41	0.39	0.38	0.36	0.33	0.36	0.41	0.43	0.50	0.56	0.60	0.63
	7	0.41	0.42	0.42	0.41	0.40	0.38	0.35	0.32	0.34	0.38	0.38	0.46	0.53	0.57	0.60	0.63
	8	0.44	0.43	0.41	0.39	0.37	0.35	0.32	0.33	0.37	0.35	0.40	0.49	0.54	0.57	0.60	0.63
	9	0.43	0.41	0.39	0.37	0.35	0.32	0.32	0.37	0.35	0.37	0.44	0.51	0.53	0.56	0.60	0.60
	10	0.42	0.39	0.38	0.35	0.32	0.32	0.37	0.35	0.37	0.40	0.46	0.51	0.52	0.55	0.57	0.58
	11	0.40	0.38	0.36	0.33	0.32	0.37	0.35	0.37	0.40	0.43	0.48	0.50	0.51	0.52	0.54	0.58
	12	0.40	0.37	0.34	0.32	0.37	0.35	0.37	0.40	0.43	0.45	0.48	0.49	0.49	0.49	0.53	0.57
	13	0.38	0.35	0.34	0.37	0.35	0.37	0.40	0.43	0.46	0.47	0.48	0.47	0.45	0.47	0.51	0.54
	14	0.34	0.34	0.39	0.36	0.37	0.40	0.43	0.46	0.47	0.47	0.47	0.43	0.42	0.43	0.45	0.48
	15	0.32	0.38	0.37	0.38	0.41	0.43	0.46	0.49	0.49	0.47	0.42	0.38	0.37	0.37	0.39	0.41
	16	0.34	0.34	0.37	0.41	0.44	0.47	0.49	0.50	0.49	0.43	0.38	0.34	0.33	0.33	0.35	0.37

c) S_i of EFI for taper ratio (Tr_a)

Recording electrodes		Stimulating electrodes 1	2	3	4	5	6	7	8	9	10	11	12	13	14	15	16
	1	0.01	0.01	0.02	0.02	0.02	0.02	0.02	0.02	0.02	0.02	0.02	0.03	0.03	0.03	0.05	0.06
	2	0.02	0.02	0.03	0.03	0.03	0.02	0.03	0.03	0.03	0.03	0.04	0.04	0.05	0.07	0.07	0.04
	3	0.04	0.04	0.04	0.04	0.04	0.05	0.05	0.05	0.06	0.07	0.08	0.09	0.11	0.10	0.08	0.06
	4	0.02	0.05	0.06	0.06	0.07	0.08	0.09	0.10	0.11	0.13	0.13	0.13	0.11	0.09	0.07	0.03
	5	0.06	0.09	0.12	0.13	0.14	0.14	0.15	0.16	0.18	0.19	0.18	0.15	0.14	0.11	0.05	0.01
	6	0.11	0.14	0.17	0.18	0.18	0.20	0.22	0.25	0.28	0.28	0.24	0.20	0.16	0.09	0.03	0.01
	7	0.13	0.17	0.20	0.22	0.24	0.26	0.30	0.34	0.34	0.29	0.27	0.22	0.14	0.06	0.02	0.01
	8	0.18	0.22	0.25	0.27	0.29	0.31	0.35	0.37	0.33	0.32	0.28	0.19	0.10	0.05	0.03	0.01
	9	0.23	0.26	0.29	0.31	0.33	0.37	0.39	0.35	0.35	0.33	0.26	0.16	0.08	0.05	0.02	0.00
	10	0.26	0.30	0.33	0.34	0.38	0.41	0.37	0.37	0.35	0.31	0.23	0.13	0.08	0.05	0.02	0.00
	11	0.30	0.34	0.36	0.39	0.42	0.39	0.38	0.36	0.32	0.28	0.19	0.12	0.09	0.05	0.01	0.00
	12	0.33	0.36	0.39	0.42	0.40	0.39	0.37	0.33	0.29	0.24	0.18	0.12	0.08	0.04	0.02	0.00
	13	0.37	0.40	0.42	0.40	0.39	0.37	0.34	0.30	0.26	0.23	0.18	0.11	0.08	0.04	0.02	0.00
	14	0.41	0.43	0.40	0.39	0.37	0.34	0.31	0.27	0.25	0.23	0.16	0.11	0.07	0.04	0.02	0.00
	15	0.46	0.42	0.40	0.37	0.35	0.32	0.28	0.25	0.24	0.20	0.15	0.09	0.07	0.04	0.01	0.00
	16	0.46	0.43	0.39	0.35	0.32	0.28	0.25	0.23	0.20	0.18	0.12	0.08	0.06	0.03	0.01	0.00

d) S_i of EFI for cochlear width (W_c)

Recording electrodes		Stimulating electrodes 1	2	3	4	5	6	7	8	9	10	11	12	13	14	15	16
	1	0.36	0.37	0.37	0.36	0.35	0.35	0.35	0.34	0.34	0.33	0.30	0.26	0.20	0.14	0.10	0.02
	2	0.37	0.37	0.35	0.34	0.34	0.34	0.34	0.35	0.34	0.31	0.27	0.23	0.19	0.13	0.03	0.07
	3	0.33	0.31	0.30	0.30	0.30	0.31	0.31	0.31	0.30	0.27	0.24	0.19	0.13	0.02	0.03	0.05
	4	0.38	0.32	0.29	0.30	0.31	0.31	0.31	0.31	0.30	0.28	0.24	0.16	0.02	0.03	0.05	0.08
	5	0.39	0.32	0.30	0.30	0.30	0.30	0.30	0.30	0.28	0.25	0.17	0.02	0.01	0.04	0.09	0.15
	6	0.35	0.29	0.27	0.27	0.27	0.26	0.25	0.23	0.18	0.11	0.01	0.00	0.03	0.09	0.16	0.20
	7	0.29	0.24	0.22	0.21	0.20	0.20	0.18	0.14	0.10	0.01	0.00	0.01	0.08	0.16	0.22	0.22
	8	0.20	0.17	0.16	0.17	0.17	0.16	0.13	0.10	0.02	0.00	0.01	0.05	0.15	0.23	0.24	0.23
	9	0.17	0.15	0.14	0.14	0.13	0.11	0.09	0.02	0.00	0.01	0.03	0.10	0.22	0.26	0.26	0.26
	10	0.14	0.13	0.12	0.12	0.10	0.08	0.02	0.01	0.01	0.03	0.06	0.17	0.25	0.27	0.29	0.29
	11	0.12	0.10	0.10	0.09	0.07	0.02	0.01	0.01	0.02	0.05	0.11	0.21	0.26	0.30	0.33	0.30
	12	0.09	0.08	0.08	0.06	0.02	0.01	0.01	0.02	0.04	0.08	0.15	0.23	0.28	0.33	0.34	0.32
	13	0.07	0.06	0.05	0.02	0.00	0.01	0.02	0.03	0.06	0.10	0.17	0.25	0.31	0.34	0.36	0.35
	14	0.05	0.04	0.01	0.00	0.01	0.01	0.03	0.05	0.08	0.11	0.19	0.29	0.35	0.39	0.42	0.42
	15	0.04	0.01	0.00	0.00	0.01	0.02	0.04	0.06	0.09	0.14	0.26	0.36	0.42	0.47	0.50	0.51
	16	0.01	0.00	0.00	0.01	0.02	0.04	0.06	0.09	0.13	0.21	0.35	0.43	0.49	0.53	0.55	0.55

e) S_i of EFI for cochlear height (h_c)

Recording electrodes		Stimulating electrodes 1	2	3	4	5	6	7	8	9	10	11	12	13	14	15	16
	1	0.01	0.01	0.01	0.01	0.01	0.01	0.01	0.01	0.01	0.00	0.00	0.00	0.01	0.01	0.02	0.11
	2	0.01	0.01	0.01	0.01	0.01	0.01	0.01	0.00	0.00	0.00	0.00	0.00	0.00	0.01	0.07	0.07
	3	0.01	0.01	0.01	0.01	0.01	0.01	0.00	0.00	0.00	0.00	0.00	0.01	0.01	0.07	0.10	0.09
	4	0.01	0.01	0.01	0.01	0.01	0.01	0.00	0.00	0.00	0.00	0.01	0.01	0.07	0.11	0.10	0.09
	5	0.01	0.01	0.01	0.01	0.01	0.00	0.00	0.00	0.00	0.01	0.01	0.08	0.12	0.11	0.09	0.06
	6	0.01	0.01	0.01	0.01	0.01	0.01	0.01	0.01	0.01	0.01	0.08	0.12	0.11	0.09	0.06	0.05
	7	0.01	0.01	0.01	0.01	0.01	0.01	0.01	0.01	0.01	0.07	0.11	0.10	0.09	0.07	0.05	0.04
	8	0.01	0.01	0.01	0.01	0.01	0.01	0.01	0.01	0.06	0.10	0.09	0.09	0.07	0.05	0.04	0.04
	9	0.01	0.01	0.01	0.01	0.01	0.01	0.01	0.05	0.09	0.09	0.08	0.07	0.05	0.04	0.04	0.03
	10	0.01	0.01	0.01	0.01	0.01	0.01	0.04	0.08	0.08	0.08	0.07	0.05	0.04	0.04	0.03	0.02
	11	0.01	0.01	0.01	0.01	0.01	0.04	0.07	0.07	0.07	0.06	0.05	0.04	0.04	0.03	0.02	0.02
	12	0.01	0.01	0.01	0.01	0.03	0.07	0.07	0.07	0.06	0.05	0.04	0.04	0.03	0.03	0.02	0.02
	13	0.01	0.01	0.01	0.03	0.06	0.06	0.06	0.06	0.05	0.04	0.04	0.03	0.03	0.02	0.02	0.02
	14	0.01	0.01	0.03	0.06	0.06	0.06	0.06	0.05	0.04	0.04	0.03	0.03	0.02	0.02	0.01	0.01
	15	0.01	0.02	0.05	0.06	0.06	0.05	0.05	0.04	0.04	0.03	0.03	0.02	0.01	0.01	0.01	0.01
	16	0.02	0.05	0.05	0.05	0.05	0.04	0.04	0.03	0.03	0.02	0.02	0.01	0.01	0.01	0.00	0.00

Supplementary Table 5| First-order Sobol sensitivity indices of EFIs for different model descriptors.

The second- and total-order sensitivity results are available from the GitHub repository in Zenodo[34].

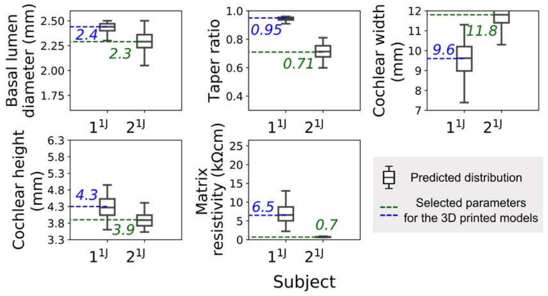

Supplementary Fig.18| The predicted distributions of the model descriptors of subjects 1^{1J} and 2^{1J}, and the selected parameters for fabricating on-demand patient-specific biomimetic cochleae.

The model descriptors were found using inverse-3PNN with the criterion that the simulated model descriptors can match the subject's off-stimulation EFI profile with a MAPE less than 6% (see Supplementary Table 8 for the MAPE thresholds used). The medians of the predicted model descriptors were then used to produce the patient-specific biomimetic cochleae. n = 1,000 samples randomly drawn from the predicted posterior distribution. The line in the box shows the median, with the box denoting the interquartile range and the whiskers denoting the ± 1.5 of the interquartile range.

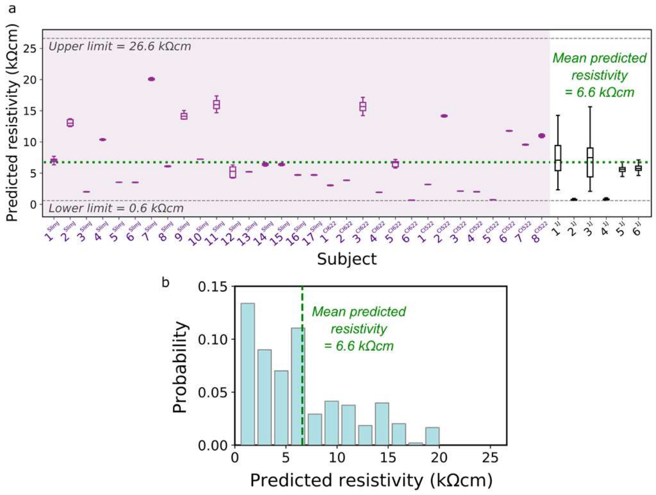

Supplementary Fig.19| 3PNN estimating patient-specific resistivity of the cochlear tissue.

- **a,** Cochlear resistivity predictions of subjects 1 – 6^{1J} (n = 6) were carried out with unknown patients' geometric descriptors, whereas the rest (n = 31) were undertaken with the information of the geometric descriptors measured from their cochlear CT scans. The MAPE threshold used in each inverse prediction can be found in Supplementary Table 8. Note that the prediction of subject 3^{CI622} was obtained with a large MAPE threshold. The line in the box shows the median, with the box denoting the interquartile range and the whiskers denoting the ± 1.5 of the interquartile range. The grey dotted lines show the reported upper and lower limits of *in vivo* human skull resistivities[2,3,5–7].

- **b,** A histogram showing the distribution of the predicted cochlear resistivity of all patients (n = 37). The mean predicted resistivity was found to be 6.6 kΩcm.

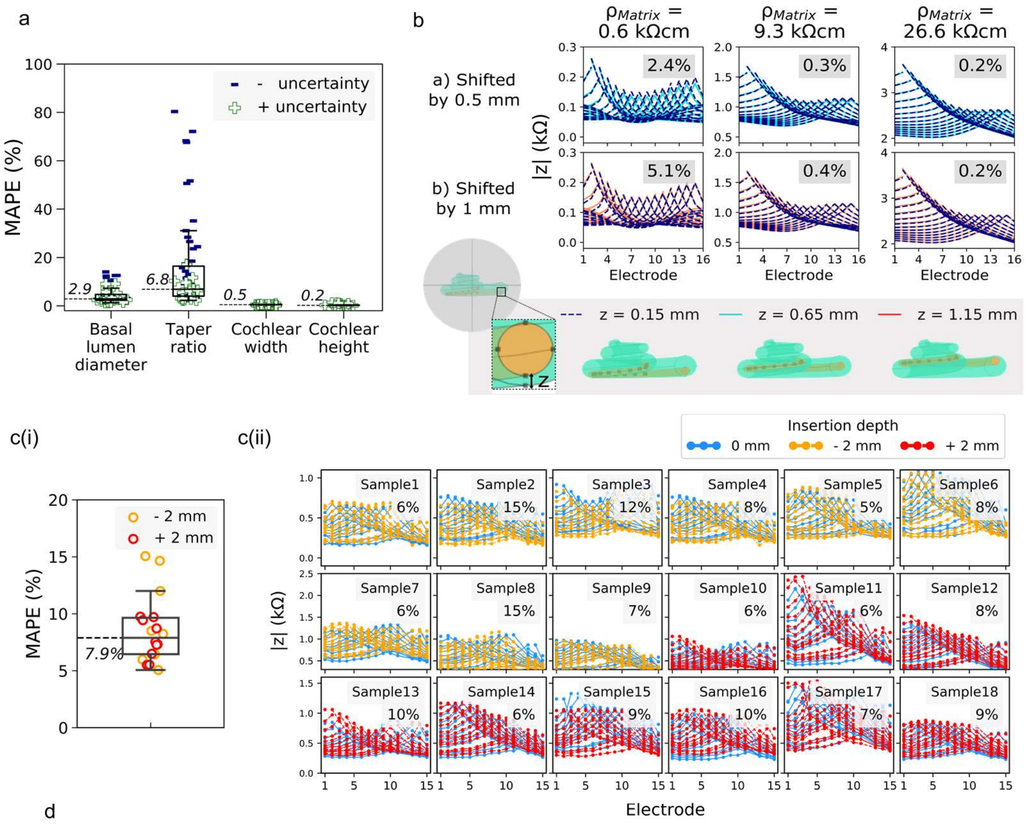

d

i) 3PNN

	Lumen diameter changed by + 0.3 mm			Lumen diameter changed by - 0.3 mm		
	0.6 kΩcm	9.3 kΩcm	26.6 kΩcm	0.6 kΩcm	9.3 kΩcm	26.6 kΩcm
Patient 1	7.9%	3.1%	2.4%	4.9%	4.7%	5.0%
Patient 2	2.7%	4.5%	5.1%	4.3%	11.7%	20.8%
Patient 3	2.0%	3.5%	3.2%	5.9%	9.1%	15.8%
**IQR**	**3.2 – 7.4 %**					
**Median**	**4.8%**					

ii) FEM

	Lumen diameter changed by + 0.3 mm			Lumen diameter changed by - 0.3 mm		
	0.6 kΩcm	9.3 kΩcm	26.6 kΩcm	0.6 kΩcm	9.3 kΩcm	26.6 kΩcm
Patient 1	7.9%	8.5%	7.6%	6.1%	10.6%	11.7%
Patient 2	4.9%	8.5%	8.2%	4.0%	10.3%	10.5%
Patient 3	5.5%	6.5%	6.1%	7.4%	10.2%	9.8%
**IQR**	**6.2 – 10.1 %**					
**Median**	**8.1%**					

Supplementary Fig.20| Sensitivity analyses assessing the effect of the potential sources of uncertainty on EFIs.

a, The effect of the uncertainty in the CT measurements of the geometric features on EFI predictions. We assume the uncertainty in the CT measurements is equal to half pixel size of the patient's CT scan. The graph shows that the MAPE between the predicted EFI using raw measurements and the predicted EFI when a geometric descriptor is subject to ± uncertainty (n = 62 predictions. 2 predictions were carried out for each patient and each prediction was subject to either + uncertainty or – uncertainty of a geometric descriptor. 31 patients were examined in total). The impact of the uncertainty in basal lumen diameter, cochlear width and cochlear height on the EFI predictions is low, while the uncertainty in taper ratio might cause an effect on the predictions with a median MAPE ~ 7%. The line in the box shows the median, with the box denoting the interquartile range and the whiskers denoting the ± 1.5 of the interquartile range.

b, Effect on simulated EFIs obtained via COMSOL when the vertical position (z-position) of the electrode array is shifted by (b) 0.5 mm and (b) 1 mm for different matrix resistivities. The value at the upper right indicates the MAPE between the z-shifted and the reference (no-shifted) cases. The geometrical features of the COMSOL model are the same as the conditions used in the model without the membrane structures in Supplementary Fig.1b(ii) and the ground was placed at the outer surface of the 8 mm radius sphere.

c, **(i)** Boxplot summarised the MAPEs of the experimental CI^{1J} EFIs acquired when there was a ± 2 mm variation in the electrode insertion depth in our 3D printed models (*n* = 18 comparisons with 9 independent comparisons examining the effect of a + 2 mm variation and 9 independent comparisons examining the effect of a - 2 mm variation). MAPE is used to quantify the deviation. Overall, an 8% median MAPE is observed. Full EFI profiles are plotted in (ii). **(ii)** Comparison of the EFIs acquired with typical insertion depth and EFIs subject to ± 2 mm insertion depth variation. The values at the upper right indicate the MAPE between the two EFI profiles. The line in the box shows the median, with the box denoting the interquartile range and the whiskers denoting the ± 1.5 of the interquartile range.

d, Effect on simulated EFIs when the cochlear lumen diameter is subject to ± 0.3 mm change obtained by **(i)** 3PNN (by changing the input values of basal lumen diameter and taper ratio) and **(ii)** FEM (by enlarging and shrinking the 3D volume of the patient's cochlea by ± 0.3 mm using Meshmixer). The values indicate the MAPEs between the simulated EFI with no geometrical change and the simulated EFI with either + 0.3 mm or – 0.3 mm change. IQR = interquartile range.

**Potential uncertainty in 3PNN**	**Approximated error on EFI (MAPE)**	
Absence of the membranous structures in the 3D printed models *(Supplementary Fig.1b)*	IQR = 2.8 – 5.0%<br>Median = 4% (*n = 5*)	
Uncertainty in patients' CT measurements *(Supplementary Fig.20a)*	BL_d	IQR = 2.3 – 4.7%, Median = 2.9% (*n = 62*)
	Tr_a	IQR = 4.0 – 16.4%, Median = 6.8% (*n = 62*)
	W_c	IQR = 0.4 – 0.5%, Median = 0.5% (*n = 62*)
	h_c	IQR = 0.2 – 0.3%, Median = 0.2% (*n = 62*)
Uncertainty in z-position of CI electrode array in cochlear lumen (shifted from 0.5 – 1 mm) *(Supplementary Fig.20b)*	IQR = 0.3% - 2.8%<br>Median = 0.4% (*n = 6*)	
Variation in CI insertion depth due to different surgical practices (± 2 mm) *(Supplementary Fig.20c)*	IQR = 6.4 – 9.6%<br>Median = 7.9% (*n = 18*)	
Geometrical discrepancy between patient cochlea and model's geometry (± 0.3 mm) *(Supplementary Fig.20d)*	IQR = 3.2 – 7.4% (3PNN) or 6.2 – 10.1% (FEM)<br>Median = 4.8% (3PNN) or 8.1% (FEM) (*n = 18*)	

Supplementary Table 6| Potential uncertainties in 3PNN, and their estimated effect on off-stimulation EFIs.

BL_d = basal lumen diameter, ρ_{matrix} = matrix resistivity, Tr_a = taper ratio, W_c = cochlear width and h_c = cochlear height. IQR = interquartile range.

Supplementary Methods

Finite element modelling of the intracochlear voltage distribution in cochlea

Finite element analyses were solved using the electric currents (ec) interface of the AC/DC module in COMSOL Multiphysics 5.5. Four types of COMSOL models were built in this study – 1) a simplified spiral model without the membrane structures, 2) a linear uncoiled model without the membrane structures, 3) a spiral model with the basilar membrane and the Reissner's membrane and 4) models using the 3D volumes of patients' cochleae (Supplementary Fig.1a(i), 1a(iii), 1b(i) and 20d(ii)).

Spiral model Creation - The spiral cochlea geometry in Supplementary Fig.1a(i) and Supplementary Fig.1b(i) was constructed according to the geometry descriptors stated in Supplementary Fig.1a(ii) and Supplementary Fig.1b(ii) captions. In detail, the cochlea geometry was formed using the sweep operation to sweep a planar circle along a parametric curve that describes the spiral trajectory of human cochleae[35]. The diameter of the planar circle was set to decrease along the parametric curve from the base to the apex linearly using the scale factor operation. Using a similar sweep operation, the geometry of the CI electrode array was built according to the geometry of the Advanced Bionics (AB) HiFocus™ 1J electrode array[21]. In the model with the membrane structures (Supplementary Fig.1b(i)), the membrane geometries were constructed in the same way using the sweep operation. The reported thicknesses of the Basilar membrane and the Reissner's membrane are 4 μm and 2.5 μm in literature[13,36]. To minimise computational errors of meshing small element, the thickness of the Basilar membrane was enlarged by a factor of 8 here. Resistivity values from literature (see Supplementary Fig.21a) were adopted to define the resistivity of the domains[1,8,11,13,14]. The permittivity effect of materials was assumed to be negligible in this study. A user-controlled mesh was applied, and a mesh convergence test was conducted to find a reasonable mesh size of the domains (Supplementary Fig.21b). The chosen mesh sizes of the domains are listed in Supplementary Fig.21a.

Linear model Creation – A linear cylinder with tapered end and the geometry of the CI electrode array were constructed according to the dimensions stated in Supplementary Fig.1a(iv) and the geometry specification of HiFocus™ 1J electrode array[21]. The mesh sizes and the resistivity of the domain were the same as those chosen in the spiral model.

Creation of models with patients' cochlear CT volumes – The CAD files of the patients' 3D volumes of cochlea are imported to COMSOL using the CAD import module. The geometry of the CI electrode array was built using the same method as described above.

In the simulations, each electrode of the implant was activated at a stimulation impulse of 800 μA one at a time. The simulated EFI profile was obtained by evaluating the surface average of voltage of each electrode plane in each electrode stimulation. The transimpedance magnitude $|z|$ was determined by dividing the surface averaged voltage by the stimulation impulse. Each COMSOL simulation takes ~ 2 min.

$$|z| = \frac{\text{Voltage, V}}{\text{Stimulation impulse, } \mathrm{I_{stim}}}$$

a

Domains	Conductivity, σ (S/m)	Sources	Mesh size (m)
Bone (sphere)	0.00118 - 0.23	Akhtari et al., 2000[8]<br>Micco et al., 2006[11]	Coarser (6.4×10^{-4})
Basilar membrane†	0.0625	Frijns et al., 1995[13]	Normal (2.88×10^{-4})
Reissner's membrane†	0.00098	Frijns et al., 1995[13]	Normal (2.88×10^{-4})
Cochlear lumen	1.79	Baumann et al., 1997[14]	Normal (2.88×10^{-4})
Platinum electrodes	8.9×10^{6}	COMSOL material library	Normal (2.88×10^{-4})
Silicone (implant)	10^{-7}	Wong et al., 2016[1]	Normal (2.88×10^{-4})

† Only present in the membranous cochlea model

b

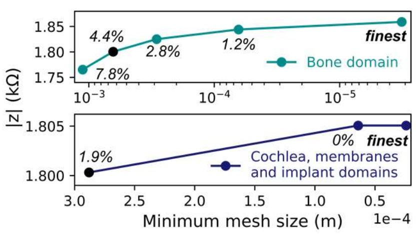

Supplementary Fig.21| Domain properties of the COMSOL models.

a, Conductivities σ and the mesh sizes of the domains of the finite element models adopted in our study.

b, Results of the mesh convergence test performed on the membranous cochlea model (Supplementary Fig.1b(i), dimensions without membranes) with conductivity of the bone domain = 0.0102 S/m. The graph shows the transimpedance magnitude $|z|$ obtained in simulating the scenario of the stimulating position at electrode 1 and the recording position at electrode 2. Simulations were carried out with different mesh sizes. We found that the computational cost can be minimized when the bone domain (sphere) and the remaining domains (cochlear lumen, implant, Basilar membrane and Reissner's membrane) were meshed with two different element sizes (top and the bottom panels). The annotations on the graph show the percentage difference relative to the result generated by the finest mesh. Result was considered as converged when the result exhibited less than 5% difference to the result generated from the finest mesh, and the corresponding mesh size was selected in our study (highlighted in black).

Embedded 3D printing of linear electro-mimetic bone matrix models

The linear models were fabricated using a similar method implemented in the fabrication of the spiral biomimetic cochlear models (see Methods – Embedded 3D printing of biomimetic cochleae). In brief, a sacrificial interconnected network, and subsequently a linear tapered sacrificial structure was embedded printed inside uncured PDMS using a 30 w/v% Pluronic F127 ink, according to the electroanatomical features stated in Supplementary Fig.1a(iv). The sacrificial Pluronic F127 ink was then removed, and the channels were filled with 1 w/v% NaCl before EFI measurements.

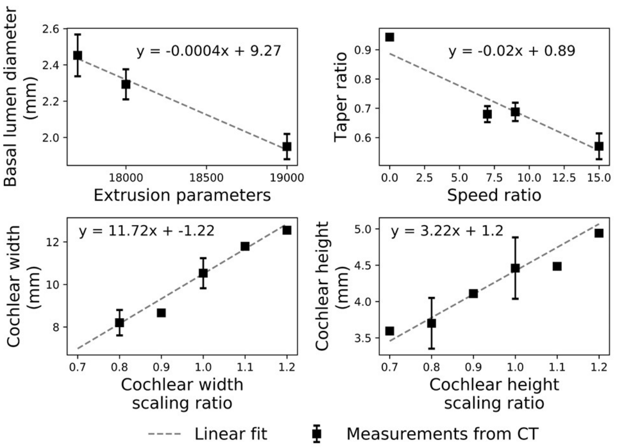

Supplementary Fig.22| Correlation of the actual dimensions of the model descriptors measured from µ-CT and the process parameters of the robotic 3D printer.

The basal lumen diameter of the 3D printed cochlea was controlled by the extrusion parameters of a bespoke robotic bioprinter, while the taper ratio was controlled by the speed ratio (the ratio of the speed of the stage when printing the apex of the cochlear lumen to the speed of the stage when printing the base). The width and the height of the printed cochlear lumen were governed by the width and the height scaling ratios of the mathematical model of human cochleae[35]. $n = 1 - 25$ measurements examined over 31 samples. Data are presented as mean values ± SD.

	ID	BL_d (mm)	Infill density	T_{ra}	W_c (mm)	h_c (mm)
**1**	101315	2.38	40	0.58	10.53	4.38
**2**	103611	2.38	40	0.70	10.53	4.38
**3**	111858	2.38	20	0.89	10.53	4.38
**4**	113438	2.38	60	0.75	10.53	3.53
**5**	114233	2.38	20	0.79	10.53	4.38
**6**	115419	2.38	20	0.75	10.53	4.38
**7**	132606	2.5	80	0.79	10.53	4.38
**8**	134101	2.5	80	0.70	10.53	4.38
**9**	141658	1.98	20	0.89	10.53	4.38
**10**	144838	2.38	20	0.89	12.66	4.38
**11**	151206	2.38	20	0.89	10.53	4.95
**12**	154018	2.38	20	0.89	10.53	3.53
**13**	154044	2.5	60	0.89	10.53	4.38
**14**	155132	2.38	60	0.79	10.53	4.38
**15**	155320	1.98	80	0.79	10.53	4.38
**16**	155745	2.38	60	0.79	8.4	4.38
**17**	160132	2.5	60	0.79	10.53	4.38
**18**	160340	1.98	40	0.89	10.53	4.38
**19**	160727	2.5	40	0.58	10.53	4.38
**20**	161812	2.38	40	0.75	10.53	4.38
**21**	162224	2.38	60	0.75	10.53	4.38
**22**	162924	1.98	40	0.79	10.53	4.38
**23**	163834	1.98	100	0.79	10.53	4.38
**24**	164032	2.5	40	0.89	10.53	4.38
**25**	164808	2.38	80	0.79	10.53	3.81
**26**	165017	2.5	60	0.75	10.53	4.38
**27**	165303	2.38	20	0.79	10.53	4.95
**28**	170130	2.5	20	0.89	10.53	4.38
**29**	170529	2.38	80	0.58	9.46	4.38
**30**	170641	2.5	40	0.79	10.53	4.38
**31**	171752	1.98	60	0.79	10.53	4.38
**32**	171916	1.98	20	0.79	10.53	4.38
**33**	172217	2.5	40	0.70	10.53	4.38
**34**	172338	2.38	60	0.58	10.53	4.38
**35**	173932	2.38	40	0.89	10.53	4.38
**36**	174214	2.38	80	0.75	10.53	4.38
**37**	174631	2.5	80	0.58	10.53	4.38
**38**	174812	2.5	60	0.58	10.53	4.38
**39**	175842	2.38	40	0.79	10.53	4.38
**40**	175858	2.38	80	0.89	10.53	4.38
**41**	175925	2.5	100	0.75	10.53	4.38
**42**	181313	2.38	80	0.79	10.53	4.38
**43**	182435	2.5	100	0.58	10.53	4.38
**44**	182512	2.38	60	0.70	10.53	3.81
**45**	182642	2.38	80	0.70	10.53	4.38
**46**	183027	2.5	80	0.89	10.53	4.38
**47**	184306	2.38	100	0.70	10.53	4.38
**48**	184732	2.38	60	0.79	10.53	4.66
**49**	185300	2.38	80	0.58	10.53	4.38
**50**	190432	2.38	60	0.79	12.66	4.38
**51**	190741	2.5	80	0.58	12.66	4.38
**52**	190950	2.5	20	0.75	10.53	4.38
**53**	191703	1.98	100	0.89	10.53	4.38
**54**	191812	2.5	80	0.58	10.53	4.95
**55**	192713	1.98	80	0.75	10.53	4.38
**56**	192815	1.98	20	0.75	10.53	4.38
**57**	193039	2.5	100	0.79	10.53	4.38
**58**	194304	2.5	20	0.79	10.53	4.38
**59**	194958	2.38	100	0.58	10.53	4.38
**60**	195345	2.5	100	0.89	10.53	4.38
**61**	201816	2.38	80	0.79	8.4	4.38
**62**	201817	2.5	60	0.70	10.53	4.38
**63**	202412	1.98	60	0.89	10.53	4.38
**64**	202511	2.38	80	0.79	9.46	4.38
**65**	202935	2.38	60	0.79	10.53	4.95
**66**	203048	2.5	20	0.58	10.53	4.38
**67**	204543	2.38	20	0.58	10.53	4.38
**68**	204710	2.38	40	0.79	11.59	4.38
**69**	204849	2.5	100	0.70	10.53	4.38
**70**	210029	2.38	60	0.70	11.59	4.38
**71**	210113	1.98	40	0.75	10.53	4.38
**72**	211311	2.38	100	0.75	10.53	4.38
**73**	212720	2.38	80	0.70	12.66	4.38
**74**	213253	2.38	80	0.79	10.53	4.95
**75**	214126	2.38	60	0.79	10.53	3.81
**76**	215112	2.5	60	0.58	10.53	4.95
**77**	220834	1.98	60	0.70	10.53	4.38
**78**	221747	2.38	60	0.70	10.53	4.38
**79**	221826	1.98	100	0.75	10.53	4.38
**80**	223926	2.38	100	0.79	10.53	4.38
**81**	225051	1.98	40	0.70	10.53	4.38
**82**	233930	1.98	60	0.75	10.53	4.38

Supplementary Table 7| Specifications of the 82 biomimetic cochleae used in 3PNN training.

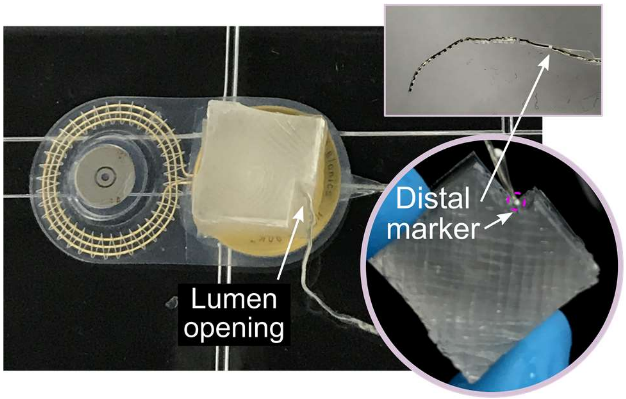

Supplementary Fig.23| Photo demonstrating the insertion of a CI electrode array in a biomimetic cochlea during EFI measurements.

The distal marker of the CI electrode array was positioned at the lumen opening of the biomimetic cochlea during EFI measurements.

	Predictions with unknown geometric features		Predictions with known geometric features		
	**Subjects**	**Final MAPE threshold (ε_f)**		**Subjects**	**Final MAPE threshold (ε_f)**
CI^{SlimJ}	Subject 1^{SlimJ}	8%†	CI^{SlimJ}	Subject 1^{SlimJ}	5% §
	Subject 2^{SlimJ}	10%†		Subject 2^{SlimJ}	10.1% §
	Subject 3^{SlimJ}	8%†		Subject 3^{SlimJ}	8% §
	Subject 4^{SlimJ}	8%†		Subject 4^{SlimJ}	8% §
	Subject 5^{SlimJ}	7%†		Subject 5^{SlimJ}	4% §
	Subject 6^{SlimJ}	8.5%†		Subject 6^{SlimJ}	5.7% §
	Subject 7^{SlimJ}	5%†		Subject 7^{SlimJ}	12.8% §
	Subject 8^{SlimJ}	6%†		Subject 8^{SlimJ}	10.8% §
	Subject 9^{SlimJ}	11%†		Subject 9^{SlimJ}	16.7% §
	Subject 10^{SlimJ}	5%†		Subject 10^{SlimJ}	7.2% §
	Subject 11^{SlimJ}	6%†		Subject 11^{SlimJ}	9.9% §
	Subject 12^{SlimJ}	5.5%†		Subject 12^{SlimJ}	10.8% §
	Subject 13^{SlimJ}	7%†		Subject 13^{SlimJ}	11.7% §
	Subject 14^{SlimJ}	3.5%†		Subject 14^{SlimJ}	5.5% §
	Subject 15^{SlimJ}	6%†		Subject 15^{SlimJ}	8.8% §
	Subject 16^{SlimJ}	6%†		Subject 16^{SlimJ}	10% §
	Subject 17^{SlimJ}	4%†		Subject 17^{SlimJ}	7.6% §
CI622	Subject 1^{CI622}	9%†	CI622	Subject 1^{CI622}	13.2% §
	Subject 2^{CI622}	7%†		Subject 2^{CI622}	13% §
	Subject 3^{CI622}	3%†		Subject 3^{CI622}	36.5% §
	Subject 4^{CI622}	11%†		Subject 4^{CI622}	15% §
	Subject 5^{CI622}	11%†		Subject 5^{CI622}	14% §
	Subject 6^{CI622}	9.5%		Subject 6^{CI622}	7.7%§
CI522	Subject 1^{CI522}	7%†	CI522	Subject 1^{CI522}	10.9% §
	Subject 2^{CI522}	11%†		Subject 2^{CI522}	13.2% §
	Subject 3^{CI522}	8%†		Subject 3^{CI522}	6.9% §
	Subject 4^{CI522}	3%†		Subject 4^{CI522}	8.3% §
	Subject 5^{CI522}	6%†		Subject 5^{CI522}	10.8% §
	Subject 6^{CI522}	6%†		Subject 6^{CI522}	11.8% §
	Subject 7^{CI522}	7.5%†		Subject 7^{CI522}	12.5% §
	Subject 8^{CI522}	6.5%†		Subject 8^{CI522}	13.7% §
CI^{1J}	Subject 1^{1J}	6%‡,*	*† Validation of inverse-3PNN (Fig.5c, Supplementary Fig.12c(ii)-13)*		
	Subject 2^{1J}	5%‡,*			
	Subject 3^{1J}	4%*	*‡ On demand patient-specific model (Fig.6b, Supplementary Fig.18)*		
	Subject 4^{1J}	10%*			
	Subject 5^{1J}	10%*	**,§ Estimation of patient-specific resistivity with unknown geometric descriptors (*) and known geometric descriptors (§) (Supplementary Fig.19)*		
	Subject 6^{1J}	6%*			

Supplementary Table 8| The final MAPE thresholds used in the inverse predictions.

Abbreviation Table

Abbreviation	Meaning
CI	Cochlear implant
CI^{1J}	Advanced Bionics (AB) HiRes 90K® implant with HiFocus™ 1J electrode array
CI^{SlimJ}	Advanced Bionics HiRes™ Ultra implant with HiFocus™ SlimJ electrode array
CI522	Cochlear™ Nucleus® Profile with slim straight electrode
CI622	Cochlear™ Nucleus® Profile Plus with slim straight electrode
MAPE	Median absolute percentage difference (%)
EFI	Electric field imaging
ε_f	Final (optimal) MAPE threshold used in inverse-3PNN
BL_d	Basal lumen diameter (Supplementary Table 1)
Tr_a	Taper ratio (Supplementary Table 1)
W_c	Cochlear width (Supplementary Table 1)
h_c	Cochlear height (Supplementary Table 1)
$\|z\|$	Transimpedance magnitude
I_{stim}	Stimulation impulse current

Supplementary References

1. Wong, P. *et al.* Development and validation of a high-fidelity finite-element model of monopolar stimulation in the implanted Guinea pig cochlea. *IEEE Trans. Biomed. Eng.* (2016).
2. Tang, C. *et al.* Correlation between structure and resistivity variations of the live human skull. *IEEE Trans. Biomed. Eng.* (2008).
3. Akhtari, M. *et al.* Conductivities of three-layer live human skull. *Brain Topogr.* (2002).
4. Briaire, J. J. & Frijns, J. H. M. Field patterns in a 3D tapered spiral model of the electrically stimulated cochlea. *Hear. Res.* (2000).
5. Fernandez-Corazza, M. *et al.* Skull modeling effects in conductivity estimates using parametric electrical impedance tomography. *IEEE Trans. Biomed. Eng.* (2018).
6. Hoekema, R. *et al.* Measurement of the conductivity of skull, temporarily removed during epilepsy surgery. *Brain Topogr.* (2003).
7. Oostendorp, T. F., Delbeke, J. & Stegeman, D. F. The conductivity of the human skull: Results of in vivo and in vitro measurements. *IEEE Trans. Biomed. Eng.* (2000).
8. Akhtari, M. *et al.* Conductivities of three-layer human skull. *Brain Topogr.* (2000).
9. Law, S. K. Thickness and resistivity variations over the upper surface of the human skull. *Brain Topogr.* (1993).
10. Saha, S. & Williams, P. A. Electric and Dielectric Properties of Wet Human Cortical Bone as a Function of Frequency. *IEEE Trans. Biomed. Eng.* (1992).
11. Micco, A. G. & Richter, C. P. Electrical resistivity measurements in the mammalian cochlea after neural degeneration. *Laryngoscope* (2006).
12. Balmer, T. W., Vesztergom, S., Broekmann, P., Stahel, A. & Büchler, P. Characterization of the electrical conductivity of bone and its correlation to osseous structure. *Sci. Rep.* (2018).
13. Frijns, J. H. M., de Snoo, S. L. & Schoonhoven, R. Potential distributions and neural excitation patterns in a rotationally symmetric model of the electrically stimulated cochlea. *Hear. Res.* (1995).
14. Baumann, S. B., Wozny, D. R., Kelly, S. K. & Meno, F. M. The electrical conductivity of human cerebrospinal fluid at body temperature. *IEEE Trans. Biomed. Eng.* (1997).
15. Nin, F. *et al.* The unique electrical properties in an extracellular fluid of the mammalian cochlea; their functional roles, homeostatic processes, and pathological significance. *Pflugers Archiv European Journal of Physiology* (2016).
16. Armin Bunde & Jan W. Kantelhardt. Diffusion and Conduction in Percolation Systems – Theory and Applications. in *Diffusion in Condensed Matter* (eds. Heitjans, P. & Jörg Kärger) 895–914 (Springer, 2005).
17. Jiang, C., de Rijk, S. R., Malliaras, G. G. & Bance, M. L. Electrochemical impedance spectroscopy of human cochleas for modeling cochlear implant electrical stimulus spread. *APL Mater.* **8**, 1–8 (2020).

18. Swanson, G. T. & Lafferty, J. F. Electrical properties of bone as a function of age, immobilization and vibration. *J. Biomech.* **5**, 261–266 (1972).

19. Lin, M. C., Herfat, S. T., Bahney, C. S., Marmor, M. & Maharbiz, M. M. Impedance spectroscopy to monitor fracture healing. in *Proceedings of the Annual International Conference of the IEEE Engineering in Medicine and Biology Society, EMBS* vols 2015-November 5138–5141 (Institute of Electrical and Electronics Engineers Inc., 2015).

20. Majdani, O. *et al.* Force measurement of insertion of cochlear implant electrode arrays in vitro: Comparison of surgeon to automated insertion tool. *Acta Otolaryngol.* (2010).

21. Advanced Bionics®. *HiRes 90K® Surgeon's Manual for the HiFocus® Helix and HiFocus® 1J Electrodes*. (2005).

22. Erixon, E., Högstorp, H., Wadin, K. & Rask-Andersen, H. Variational anatomy of the human cochlea: Implications for cochlear implantation. *Otol. Neurotol.* (2009).

23. Avci, E., Nauwelaers, T., Lenarz, T., Hamacher, V. & Kral, A. Variations in microanatomy of the human cochlea. *J. Comp. Neurol.* (2014).

24. Meng, J., Li, S., Zhang, F., Li, Q. & Qin, Z. Cochlear size and shape variability and implications in cochlear implantation surgery. *Otol. Neurotol.* (2016).

25. Mori, M. C. & Chang, K. W. CT analysis demonstrates that cochlear height does not change with age. *Am. J. Neuroradiol.* (2012).

26. Smith, A. & Mason, A. K. Cost estimation predictive modeling: Regression versus neural network. *Eng. Econ.* (1997).

27. Vanpoucke, F. J., Zarowski, A. J. & Peeters, S. A. Identification of the impedance model of an implanted cochlear prosthesis from intracochlear potential measurements. *IEEE Trans. Biomed. Eng.* (2004).

28. Suesserman, M. F. & Spelman, F. A. Lumped-Parameter Model for In Vivo Cochlear Stimulation. *IEEE Trans. Biomed. Eng.* (1993).

29. Strelioff, D. A computer simulation of the generation and distribution of cochlear potentials. *J. Acoust. Soc. Am.* (1973).

30. Kohavi, R. A Study of Cross-Validation and Bootstrap for Accuracy Estimation and Model Selection. *Int. Jt. Conf. Artif. Intell.* (1995).

31. Advanced Bionics®. *TECHNICAL SPECIFICATIONS - HiRes™ Ultra Cochlear Implant with the HiFocus™ SlimJ and HiFocus™ Mid-Scala Electrodes*. (2017).

32. Cochlear®. *Technical Specifications - Cochlear™ Nucleus® Profile™ Plus with Slim Straight Electrode (CI622)*. (2019).

33. Sobol, I. M. Global sensitivity indices for nonlinear mathematical models and their Monte Carlo estimates. *Math. Comput. Simul.* (2001).

34. Lei, I. M. *et al.* 3D printed biomimetic cochleae and machine learning co-modelling provides clinical informatics for cochlear implant patients. *Zenodo.* https://doi.org/10.5281/zenodo.5353394 (2021).

35. Pietsch, M. *et al.* Spiral Form of the Human Cochlea Results from Spatial Constraints.

Sci. Rep. (2017).

36. Harada, Y. & Harada, Y. Reissner's membrane. in *Atlas of the Ear* 165–169 (Springer Netherlands, 1983).
