# Supplementary material for: 3D printed biomimetic cochleae and machine learning co-modelling provides clinical informatics for cochlear implant patients
Source: Nat Commun. 2021 Oct 29;12:6260. doi: 10.1038/s41467-021-26491-6 (PMC8556326; doi:10.1038/s41467-021-26491-6)
Supplement: Supplementary file 3 — Description of Additional Supplementary Files [file 41467_2021_26491_MOESM3_ESM.docx]

Description of Additional Supplementary Files

Title: Supplementary Video 1| Micro-CT volumes of an exemplar 3D printed biomimetic cochlea.

Description: Video shows the rendered 3D volumes of a 3D printed electro-mimetic bone matrix, a printed cochlear lumen embedded in an electro-mimetic bone matrix and a printed cochlear lumen with a CI electrode array inserted.

Title: Supplementary Video 2| Embedded printing methodology for fabricating biomimetic cochleae.

Description: An electro-mimetic bone matrix was first created by embedded printing Pluronic F127 sacrificial microchannels in a bath of uncured PDMS (coloured with sodium fluorescein). Following this, a sacrificial tapered spiral was embedded printed inside the uncured electro-mimetic bone matrix with Pluronic F127 for creating the biomimetic cochlear lumen. The printed spiral was coloured with sodium fluorescein, and the sacrificial microchannels were uncoloured in the second step of the video. The size of the container in the video was 3x3x3.5 cm.
