# Supplementary material for: 3D printed biomimetic cochleae and machine learning co-modelling provides clinical informatics for cochlear implant patients
Source: Nat Commun. 2021 Oct 29;12:6260. doi: 10.1038/s41467-021-26491-6 (PMC8556326; doi:10.1038/s41467-021-26491-6)
Supplement: Supplementary file 6 — Reporting Summary [file 41467_2021_26491_MOESM6_ESM.pdf]

nature research

Corresponding author(s): Yan Yan Shery Huang
Manohar Bance

Last updated by author(s): Sep 1, 2021

Reporting Summary

Nature Research wishes to improve the reproducibility of the work that we publish. This form provides structure for consistency and transparency in reporting. For further information on Nature Research policies, see our Editorial Policies and the Editorial Policy Checklist.

Statistics

For all statistical analyses, confirm that the following items are present in the figure legend, table legend, main text, or Methods section.

n/a	Confirmed	
☐	☒	The exact sample size (*n*) for each experimental group/condition, given as a discrete number and unit of measurement
☐	☒	A statement on whether measurements were taken from distinct samples or whether the same sample was measured repeatedly
☒	☐	The statistical test(s) used AND whether they are one- or two-sided<br>*Only common tests should be described solely by name; describe more complex techniques in the Methods section.*
☒	☐	A description of all covariates tested
☐	☒	A description of any assumptions or corrections, such as tests of normality and adjustment for multiple comparisons
☐	☒	A full description of the statistical parameters including central tendency (e.g. means) or other basic estimates (e.g. regression coefficient) AND variation (e.g. standard deviation) or associated estimates of uncertainty (e.g. confidence intervals)
☒	☐	For null hypothesis testing, the test statistic (e.g. *F*, *t*, *r*) with confidence intervals, effect sizes, degrees of freedom and *P* value noted<br>*Give P values as exact values whenever suitable.*
☐	☒	For Bayesian analysis, information on the choice of priors and Markov chain Monte Carlo settings
☐	☒	For hierarchical and complex designs, identification of the appropriate level for tests and full reporting of outcomes
☒	☐	Estimates of effect sizes (e.g. Cohen's *d*, Pearson's *r*), indicating how they were calculated

Our web collection on statistics for biologists contains articles on many of the points above.

Software and code

Policy information about availability of computer code

Data collection	All EFI profiles of the 3D printed models, and the patient clinical EFIs acquired in our centre (Emmeline Centre for Hearing Implants in Cambridge, UK) were measured with either AB Volta version 1.1.1 (research only) or Custom Sound® EP 5.1 (with research option). The CT scans of the 3D printed models were acquired with Scout-and-Scan™ Control. Impedance measurements were acquired using SMaRT 3.0.1 impedance measurement software.
Data analysis	Python 3.7.6, ImageJ 2.0.0-rc-69/1.52p, Slicer 4.10.2, SALib 1.4.0.1, Tensorflow 2.1.0, PINTS 0.3.0, COMSOL Multiphysics 5.5 and the codes of the neural network model are available from https://doi.org/10.5281/zenodo.5353394.

For manuscripts utilizing custom algorithms or software that are central to the research but not yet described in published literature, software must be made available to editors and reviewers. We strongly encourage code deposition in a community repository (e.g. GitHub). See the Nature Research guidelines for submitting code & software for further information.

Data

Policy information about availability of data

All manuscripts must include a data availability statement. This statement should provide the following information, where applicable:

- Accession codes, unique identifiers, or web links for publicly available datasets
- A list of figures that have associated raw data
- A description of any restrictions on data availability

The raw data of the 3PNN validation, Sobol sensitivity analysis, the stimulus spread trend, the resistivity prediction and the uncertainty sensitivity analyses have been deposited in Github (https://github.com/chonlei/3PNN) and in Zenodo under accession code (https://doi.org/10.5281/zenodo.5353394). Other data generated in this study are provided in the Source Data file. The clinical data will be made available upon reasonable request to the corresponding authors and in compliance with the relevant ethical guideline.

April 2020

Field-specific reporting

Please select the one below that is the best fit for your research. If you are not sure, read the appropriate sections before making your selection.

[x] Life sciences   [ ] Behavioural & social sciences   [ ] Ecological, evolutionary & environmental sciences

For a reference copy of the document with all sections, see nature.com/documents/nr-reporting-summary-flat.pdf

Life sciences study design

All studies must disclose on these points even when the disclosure is negative.

Sample size	82 samples were used to create the neural network model. Sample size were chosen on the basis of the accuracy of the model and the experimental cost. The 10-fold cross-validation in our study showed that 82 samples are satisfactory to achieve a reasonably good level of accuracy.<br><br>31 anonymous clinical EFI profiles with paired CT scans were used to validate the accuracy of the neural network model. These profiles were chosen to ensure their representativeness of the patient population variation in EFI by comparing them with 97 clinical data.
Data exclusions	HiFocus 1J electrode array EFI data from electrodes 12 and 16, and Cochlear Corporation Nucleus® CI522 data from electrodes 1,12,13,15,18 and 22 were excluded in this study due to their unavailability, but this does not affect the measurements of other electrodes and the general shape of the EFI or transimpedance profiles.
Replication	The impedance measurements of the electro-mimetic bone matrices were repeated at least 3 times. EFI measurement in each 3D printed cochlear models was repeated 3 times. All attempts at replication were successful. 3D printed models were tested with different cochlear implant electrodes (HiFocus 1J electrode array, HiResTM Ultra HiFocusTM SlimJ electrode and Cochlear Corporation Nucleus® CI522) to ensure the general shapes of EFIs measured with different implants in the same model are the same (n = 15 in total). The applicability of the neural network model on predicting EFIs of different cochlear implant electrodes were validated with 4 electrode types (n = 15 with data from 3D printed models and n = 31 with clinical data). The correlations between the printing parameters and the actual features were calibrated.
Randomization	This study did not involve group allocation, hence randomisation of participants did not apply.
Blinding	This study did not involve group allocation, hence blinding did not apply in this study.

Reporting for specific materials, systems and methods

We require information from authors about some types of materials, experimental systems and methods used in many studies. Here, indicate whether each material, system or method listed is relevant to your study. If you are not sure if a list item applies to your research, read the appropriate section before selecting a response.

Materials & experimental systems

n/a	Involved in the study	
[x]	[ ]	Antibodies
[x]	[ ]	Eukaryotic cell lines
[x]	[ ]	Palaeontology and archaeology
[x]	[ ]	Animals and other organisms
[ ]	[x]	Human research participants
[x]	[ ]	Clinical data
[x]	[ ]	Dual use research of concern

Methods

n/a	Involved in the study	
[x]	[ ]	ChIP-seq
[x]	[ ]	Flow cytometry
[x]	[ ]	MRI-based neuroimaging

Human research participants

Policy information about studies involving human research participants

Population characteristics	In total, 128 clinical EFI profiles (also known as transimpedance matrices) were used in this study. Of the 128 profiles, 91 profiles were acquired independently by Advanced Bionics® (without paired CT scan information), and the rest were obtained from 37 anonymous patients (31 with paired CT scan data and 6 without paired CT data) who have undergone cochlear implantation at the Emmeline Centre for Hearing Implants in Cambridge, UK. Out of the 37 profile data acquired in our centre, 6 profiles were acquired from the Advanced Bionics HiRes 90K® implant with HiFocus 1J electrode array, 17 profiles from the Advanced Bionics HiRes Ultra implant with HiFocus SlimJ electrode array, 6 profiles from the Cochlear Nucleus® Profile Plus with slim straight electrode CI622, and 8 profiles from the Cochlear Nucleus® Profile with slim straight electrode CI522. The data were anonymised, hence no personally identifiable information (e.g. gender and age) were available.
Recruitment	Retrospective data from cochlear implant patients were used, hence recruitment did not apply. The data tested here are within two standard deviations of the mean patient EFIs (derived from 97 patients' EFIs). They were randomly selected with no bias.
Ethics oversight	The use of anonymous patient EFI profiles with or without paired CT scans in our study was approved by the University of Cambridge Human Biology Research Ethics Committee (HBREC.2019.42) and the Cambridge Biomedical Research Centre (Ref: A095451).

Note that full information on the approval of the study protocol must also be provided in the manuscript.
